# A hierarchy in clusters of cephalopod mRNA editing sites

**DOI:** 10.1038/s41598-022-07460-5

**Published:** 2022-03-02

**Authors:** Mikhail A. Moldovan, Zoe S. Chervontseva, Daria S. Nogina, Mikhail S. Gelfand

**Affiliations:** 1 Skolkovo Institute of Science and Technology, Bolshoy Boulevard 30, bld. 1, Moscow, Russia 121205; 2 A.A.Kharkevich Institute for Information Transmission Problems (RAS), Bolshoy Karetny Per. 19, bld.1, Moscow, Russia 127051; 3 Faculty of Bioengineering and Bioinformatics, M.V. Lomonosov Moscow State University, Leninskie Gory 1, Moscow, Russia 119991

**Keywords:** Genome informatics, Molecular biology, Transcription

## Abstract

RNA editing in the form of substituting adenine with inosine (A-to-I editing) is the most frequent type of RNA editing in many metazoan species. In most species, A-to-I editing sites tend to form clusters and editing at clustered sites depends on editing of the adjacent sites. Although functionally important in some specific cases, A-to-I editing usually is rare. The exception occurs in soft-bodied coleoid cephalopods, where tens of thousands of potentially important A-to-I editing sites have been identified, making coleoids an ideal model for studying of properties and evolution of A-to-I editing sites. Here, we apply several diverse techniques to demonstrate a strong tendency of coleoid RNA editing sites to cluster along the transcript. We show that clustering of editing sites and correlated editing substantially contribute to the transcriptome diversity that arises due to extensive RNA editing. Moreover, we identify three distinct types of editing site clusters, varying in size, and describe RNA structural features and mechanisms likely underlying formation of these clusters. In particular, these observations may explain sequence conservation at large distances around editing sites and the observed dependency of editing on mutations in the vicinity of editing sites.

## Introduction

The mRNA editing process, where an adenine is substituted by inosine (A-to-I editing), is a widespread mechanism of transcriptome diversification in metazoans^1–5^. Inosine is recognized by the cellular machinery as guanine^6–12^, and hence the proteins translated from an edited transcript may be re-coded, thus contributing to the proteome diversity^12–14^. A-to-I editing is performed by the family of ADAR enzymes, and mutations corrupting ADAR may cause reduction of fitness in model organisms and disease in humans^10,14–18^.

**Figure 1:**
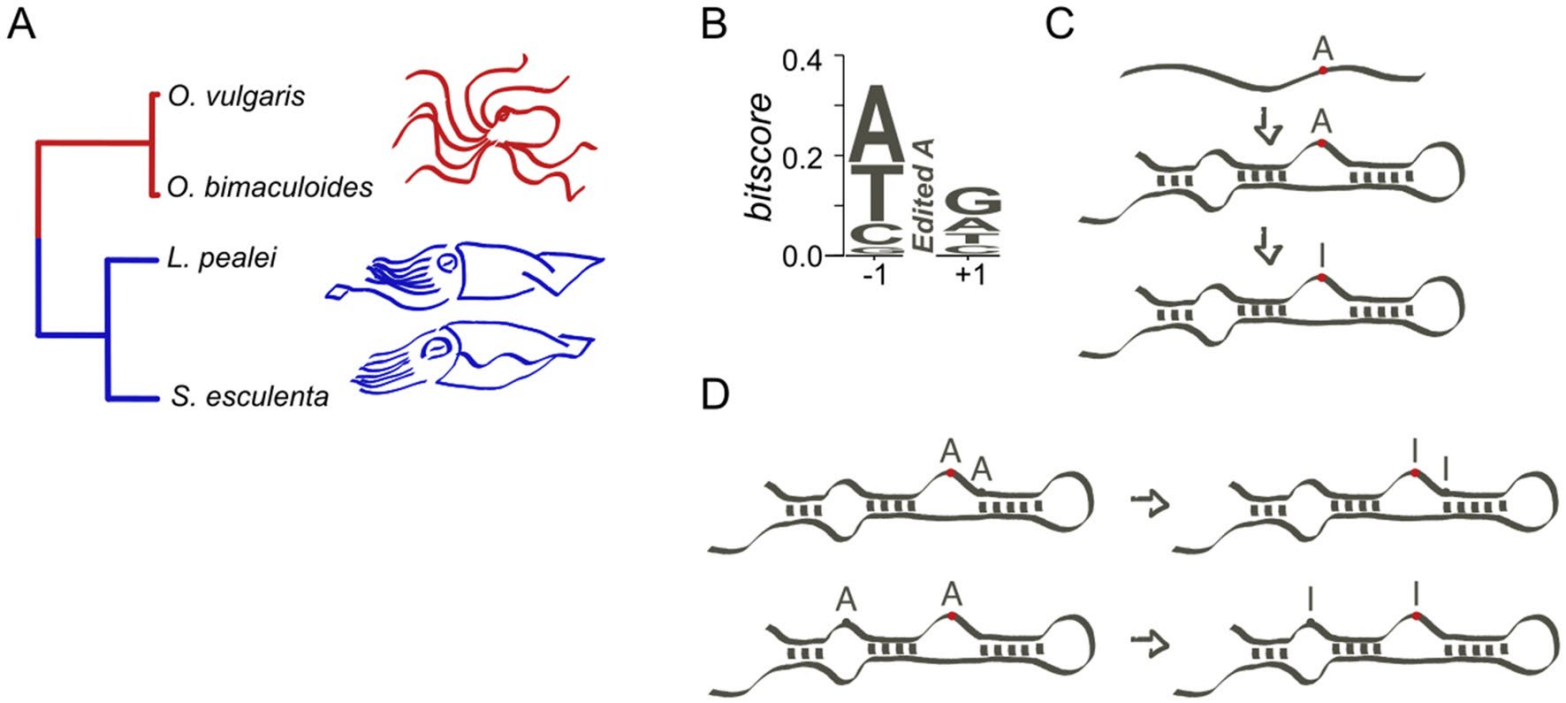
(**A**) Phylogenetic tree of four mollusks (octopuses *Octopus vulgaris* and *O. bimaculoides*, squid *Loligo pealei*, and cuttlefish *Sepia esculenta*) considered in this study. The tree has been taken from TimeTree^48^. (**B**) Sequence context of coleoid A-to-I editing sites. (**C**) ADAR enzymes performing A-to-I editing require secondary RNA structures. (**D**) Editing at closely and at distantly located sites. See the text for details.

Still, A-to-I editing sites are rare in coding regions of most genomes studied so far, with only minor fractions of them being conserved or functionally important^3,19–23^. However, in coleoids (soft-bodied cephalopods, Fig. 1A), not only A-to-I editing is frequent, but is also more functionally important than in other studied lineages, i.e. mammals and *Drosophila*^13,14,24^. Editing in coleoids involves up to 1% of all adenines in the transcriptomes and has been suggested to play an important role in proteome diversification, allowing for responses to many environmental cues, such as phenotypic adjustments to low temperatures^13,14,25^. Along with that, editing sites could have an evolutionary value by rescuing deleterious G-to-A substitutions^26,27^ or by providing heritable phenotypes selection can act upon, thus enhancing the rate of adaptation^28,29^.

To edit transcripts, ADAR enzymes require specific features of the sequence around editing sites^2,4,5,12,13,30^. Along with the edited adenine itself, a specific nucleotide context is required at positions ± 1 relative to the edited adenine. However, the consensus at these positions is rather weak^13,24,28,31^. The ADAR enzymes also require edited adenines to be located in RNA helices, which may form complex structures spaning over 1 kb of linear nucleotide sequence^2,5,32–34^. Thus, editing at individual sites may be influenced by distant loci, which has been shown directly by the edQTL analysis^35^. However, on average, the span of regions affecting editing at a particular site is about 200–400 nt^13^, as shown by edQTL studies and analysis of sequence conservation in regions around editing sites in *Drosophila*^35^ and coleoids^13^, respectively.

Nonetheless, the ADAR requirements on sequence and structure to edit a particular site are rather weak, yielding multiple weakly edited adenines in every studied transcriptome. Consequently, editing sites have been proposed to form constantly at random points of the genome, especially in structured RNA segments^36^. Adjacently located edited adenines tend to be edited simultaneously^32,37–40^. In human and *Drosophila*, such correlations are mainly due to the involvement of such sites in the same secondary RNA structures. Additionally, editing sites located in coding regions are clustered for *Drosophila* and leaf-cutter ants, with clusters arbitrarily defined as groups of editing sites where adjacent sites are located at most at 30–50 nt from each other^41,42^.

Clustering of editing sites has been extensively studied in tandem, differently oriented Alu repeats, where formation of Alu-Alu double helices is common^34,43–45^. Editing of Alu repeats has been hypothesized to protect against negative effects of Alu repeats on the organism’s fitness^14^ and Alu sequences may be used as indicators of the general editing activity in tissues^34^. Extensive editing at Alu sequences also allows for the analyses of subtle features of ADAR-mediated editing such as the correlated editing at specific sites^34^ or establishment of preferential sequence of editing events along Alu-containing transcripts^45^.

In coding sequences, clusters of A-to-I editing sites are also present and abundant, with clustered editing sites being on average more conserved and heavily edited than their individual counterparts^13,37^. The enhanced conservation of clustered editing sites, their distance-dependent linkage, and dependencies of editing at one site on editing at another^37,45^ suggest the importance not only of A-to-I editing per se, but also of dynamics of the editing process, so that editing tends to occur simultaneously at a multitude of sites in a given transcript. This hypothesis is supported by the observation that non-synonymous editing sites in protein-coding sequences are more clustered than synonymous ones^37^.

By having a large number of A-to-I editing sites, coleoids are a perfect model for studying subtle evolutionary and statistical features of RNA editing. One relevant question is posed by the possible structure of A-to-I editing clusters and the processes underlying formation of clusters with specific structures. As coleoid editing sites demonstrate same contextual features and secondary RNA structure requirements as mammalian or *Drosophila* editing sites (Fig. 1B,C)^13,28,36^, studying coleoids as a convenient model we may enhance our understanding of the ADAR action in general and of the evolutionary and functional mechanisms involved in the emergence of new editing sites.

Here, we rely on four coleoid transcriptomes to show that the level of association between A-to-I editing at individual sites in coding regions strongly depends on the distances between the sites. The underlying intuition here is as follows: closely located adenines should be similar in terms of local RNA structure, and if one of them is edited, the other one is more likely to have the necessary prerequisites for ADAR-mediated editing. Hence, we expect more closely located adenines to be edited simultaneously with a higher probability than more distantly located ones. with the highest correlation observed for immediately adjacent edited adenines (Fig. 1D).

By applying multiple and diverse approaches to analyze the distribution of editing sites along transcripts, we identified three distinct types of clusters of coleoid editing sites with sharply different characteristic size ranges. Analyzing local RNA structural features, we observe a tendency of editing sites to be located in putative loops, mismatches or bulges in secondary RNA structures, in agreement with observations of individual A-to-I editing sites that form A-C mismatches in RNA helices^5,33,46,47^. In addition, we show that correlated editing in coding regions strongly contributes to transcriptome diversity driven by ADAR-mediated editing in general and that editing in clusters generally occurs in the 3’-to-5’ direction.

## Results

### Correlated editing

**Figure 2:**
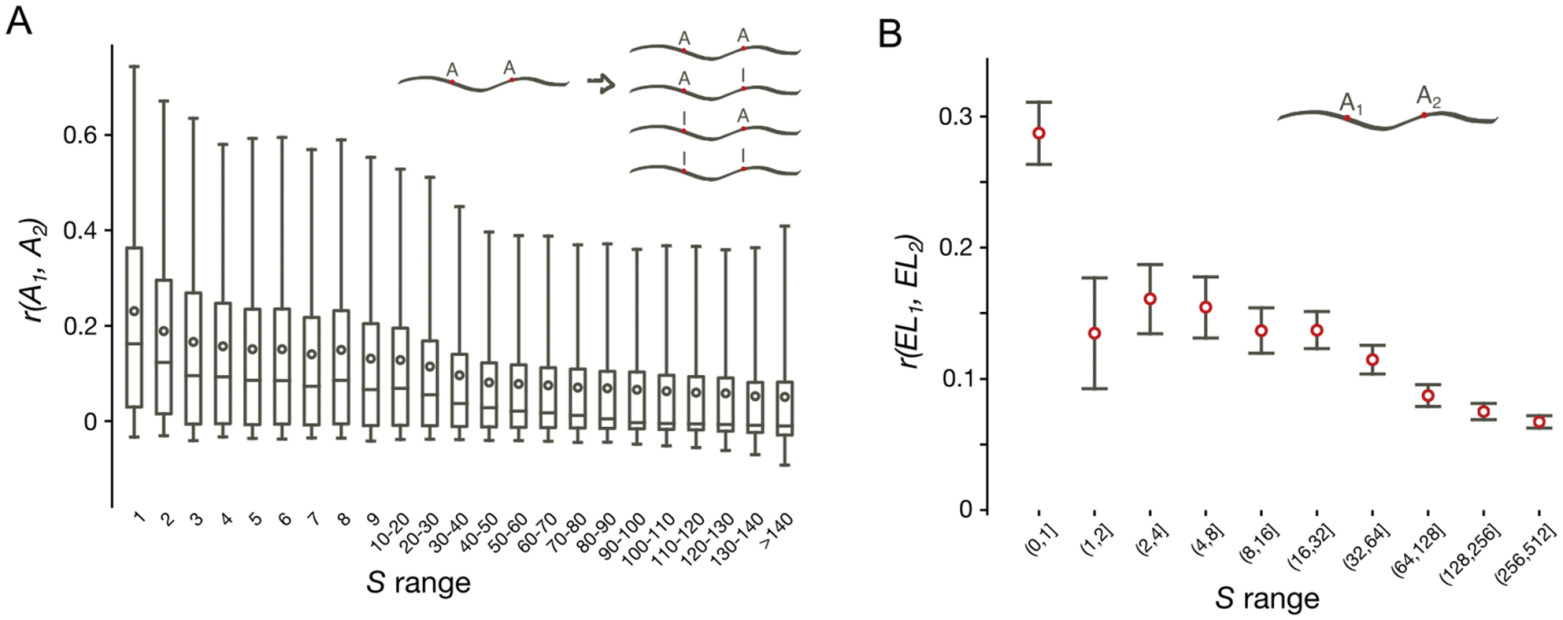
Correlations between various properties of editing sites. (**A**) Distributions of correlation coefficients of *O. vulgaris* editing (*r*) at two sites with respect to the distances between sites (*S*). Boxes represent quartiles, red circles represent the means and the grey lines (whiskers) indicate 95% two-sided confidence intervals of distributions. (**B**) Dependence of correlations of ELs on the *S* distance, *O. vulgaris* dataset. Red circles mark values of correlation coefficients and grey lines represent Bonferroni corrected 95% two-sided confidence intervals obtained from the *t*-distribution.

In model metazoan species, editing may be correlated if the sites are located sufficiently close to each other^37^. The unusually large numbers of coleoid editing sites allowed us to assess the interplay between co-occurrences of editing states and the distances between editing sites at the single-nucleotide resolution. In our study, we used the available trascriptomes and editing site sets for four coleoids—two octopuses *Octopus vulgaris* and *O. bimaculoides*, *Sepia esculenta* (cuttlefish), and *Loligo pealei* (squid)^13^. We used raw RNAseq data (Supplementary Table S1) to calculate the correlation of edited states for each pair of editing sites separated by at most the distance equal to read lengths in our dataset (~ 100–150 nt) (Supplementary Table S1). The correlation coefficients for a pair of edited adenines *E*_*i*_ and *E*_*j*_ given the RNAseq read mapping to transcripts is defined as in^37^ (Fig. 2A, Supplementary Figs. S1, S2): $r\left({E}_{i},{E}_{j}\right)= ({f}_{i,j}^{AA}{f}_{i,j}^{II}- {f}_{i,j}^{AI}{f}_{i,j}^{IA})/\sqrt{{f}_{i}^{A}{f}_{i}^{I}{f}_{j}^{A}{f}_{j}^{I}}$, where ${f}_{i,j}^{{N}_{1}{N}_{2}}$ are frequencies of co-occurrences of observed nucleotides ${N}_{1}$ and ${N}_{2}$ (A or I/G) at positions *i* and *j* in the RNAseq read data, and ${f}_{i}^{N}$ are frequencies of nucleotide *N* in the read mapping data at position *i*. We compared the distributions of $r\left({E}_{i},{E}_{j}\right)$ for different inter-site distances, which we refer to as the *S* values, *S* defined as *j* − *i* (Fig. 2A). The correlations were on average higher for immediately adjacent sites, with mean $r\left({E}_{i},{E}_{j}\right)$ values further decreasing with the increase of the *S* distance, consistent with observations in ref.^37^.

The editing level (EL) of an A-to-I editing site is defined as the percentage of mapped reads in a sample containing inosine (read as guanine) at the considered site. As the editing levels of most sites are rather low (< 10%), one could speculate that the bulk of associations is lost in the above analyses due to missed low-EL sites that could not be retrieved from the data^31^. Indeed, if we consider sites with EL ≥ 5% (Supplementary Fig. S2a), the average $r\left({E}_{i},{E}_{j}\right)$ values increase almost twofold, reaching 0.43 for *S* = 1. To check whether higher $r\left({E}_{i},{E}_{j}\right)$ values are not simply a property of efficiently edited sites, we calculated the $r\left({E}_{i},{E}_{j}\right)$ distributions for sites with the EL ≥ 10% and obtained only slightly larger $r\left({E}_{i},{E}_{j}\right)$ values, as compared to sites with EL ≥ 5% (Supplementary Fig. S2b). Thus, the association between the A-to-I editing events is indeed strong, especially for adjacently located editing sites.

To check whether the editing state co-occurrence manifests as similarities between ELs, we assessed the correlations between the ELs at individual sites for a series of *S* values (Fig. 2B, Supplementary Fig. S3). For immediately adjacent editing sites (*S* = 1), this correlation turned out to be on average twofold larger than for any other *S* (*p* < 0.001, the *t*-test). If adjacent sites are not considered, the correlations in ELs only slightly depend on *S*, being significant (*p* < 0.05, the *t*-test) even for quite distantly located sites (*S* > 500). Non-zero EL correlations at very large distances may be explained by some transcripts being edited to a higher overall degree than other transcripts. An alternative explanation is as follows. The general variance of ELs in the transcriptome may be decomposed into two summands: the between-transcript variance and the within-transcript variance, the former being the variance of the mean EL values in transcripts, and the latter being the variance of the deviations of ELs from the means in each transcript. If the between-transcript variance is non-zero due to, e.g. low average numbers of editing sites per transcript yielding the estimates of means with high variance, we would observe a baseline correlation for any *S* value, which is simply not defined for sites located in different transcripts.

In theory, correlated editing at different sites may enhance the transcriptome diversity defined as the number of possible states with respect to editing. So if there is one editing site, which can be either in inosine or adenine state, this number would be 2, if there are two sites—4, etc.^45^. Here, the increase in transcriptome diversity due to correlated editing may seem counterintuitive, as dependencies in editing events should generally decrease the possible numbers of transcript variants in a given cell. However, the number of possible transcriptome states in coleoids even under complete linkage of editing events is still astronomically large, and thus hardly represents a bottleneck of transcriptome diversity: on average, about 8000 coleoid genes are edited, which yields 2^8000^, or 10^2408^ transcriptome states.

An alternative approach here could be to assess the variance in transcriptome and proteome generated by editing. If we, following the definition of between-site correlation, define the variance in an editing site *i* as ${f}_{i}^{A}{f}_{i}^{I}$ and the covariance between two sites *i* and *j* as ${f}_{i,j}^{AA}{f}_{i,j}^{II}- {f}_{i,j}^{AI}{f}_{i,j}^{IA}$, we can calculate the net variance generated by editing to be up to 111 in transcriptomes and up to 92 in proteomes (Supplementary Table S2). In the context of populations, such variance can be generated by 888 and 736 two-allele polymorphisms with minor allele frequencies of 0.5 without dominance, which is rather large. Moreover, we find almost half of this variance to be explained by correlated editing at pairs of sites, namely, up to 46.3% of the transcriptome variance and up to 46.5% of the proteome variance. These percentages likely represent lower bounds, as covariances incorporated in this analysis have had to satisfy stringent statistical criteria, otherwise they have been set to zero (see Suppl. Methods).

### Dense editing site clusters (adjacent adenines)

Notably, the correlation between ELs is by far the highest for immediately adjacent editing sites with *S* = 1 (Fig. 2B). We consider these sites separately and refer to them as *dense editing site clusters* (DCs) in the general case, and as *paired editing sites* if there are only two adenines per cluster. The observed enhanced positive correlation of editing site co-occurrence for dense clusters (Fig. 2) hints at editing at a focal site being dependent on editing at the immediately adjacent adenine. This could lead to overrepresentation of DCs in the coleoid transcriptomes.

**Figure 3:**
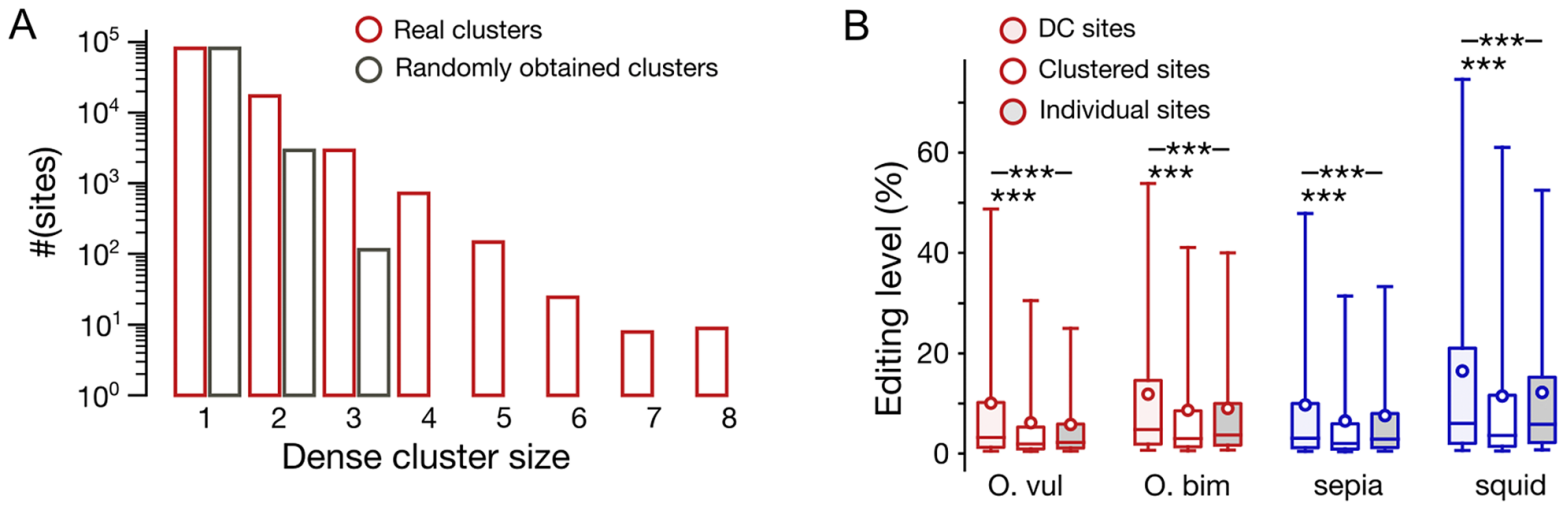
Properties of densely clustered A-to-I editing sites. (**A**) Histogram of dense cluster sizes (nt) for the real *O. vulgaris* editing site dataset (red) and a matching random dataset (grey). (**B**) Comparison of editing levels in densely clustered (*S* = 1, red and blue filled boxes), not densely clustered (1 < *S* < 100, white boxes) sites, and individual sites (*S* ≥ 100, grey-filled boxes). Three asterisks mark statistical significance of the differences in means (*p* < 0.001, the Mann–Whitney U-test).

To check whether DCs are indeed overrepresented, we calculated the numbers of sites in DCs separately for each DC size across the coleoid transcriptomes (Fig. 3A, Supplementary Fig. S4). As controls, we randomly selected adenines with and without regard to the local trinucleotide context (see Supplementary Methods). The results obtained for the two control sets did not differ (Supplementary Fig. S4). For all DC sizes, which ranged from two to eight consecutive adenines, the site count in the real datasets was larger than that in the control datasets, the effect being stronger for DCs with larger numbers of adenines (Fig. 3A, Supplementary Fig. S4A, Supplementary Fig. S5).

Given the observed stronger association of editing at heavily edited adenines compared to that of weakly edited ones (Fig. 2A, Supplementary Fig. S2), one would expect enhanced editing levels of adenines in DCs. However, the enhanced levels of editing at clustered sites should also be taken into account. Thus, following ref.^37^, we have divided editing sites into clustered sites with the between-site distance smaller than 100nt and individual sites, for which no editing is observed in the 100nt vicinity. To disentangle effects on editing conveyed by < 100nt proximity and by location of sites in DCs, we further divided the set of clustered adenines into editing sites located in DCs and non-DC clustered sites, and compared the distributions of ELs in all three resulting categories of sites (Fig. 3B). The average ELs of sites in DCs were up to 1.67-fold larger than those of individual sites (*p* < 2.4 × 10^−7^, the Mann–Whitney U-test) and up to 1.59-fold larger than average ELs of non-DC clustered sites (*p* < 9.8 × 10^−201^, the Mann–Whitney U-test). Accordingly, the fraction of heavily edited sites (EL > 50%) in DCs is up to 3.42-fold larger than that in individual sites (*p* < 1.96 × 10^−45^, the χ^2^ contingency test) and up to twofold larger than in non-DC clustered sites (*p* < 2.26 × 10^−6^, the χ^2^ contingency test). Interestingly, we did not observe consistently significant differences between ELs at individual versus non-DC clustered sites, which shows that the effects of clustering on EL observed in^37^ are largely conferred by densely clustered sites. However, non-DC clustered sites differ from individual ones when the fraction of heavily edited sites is considered, which is up to 1.65-fold higher in non-DC clustered sites (*p* < 1.7 × 10^−4^, the χ^2^ contingency test).

### Medium-range clusters of editing sites

**Figure 4:**
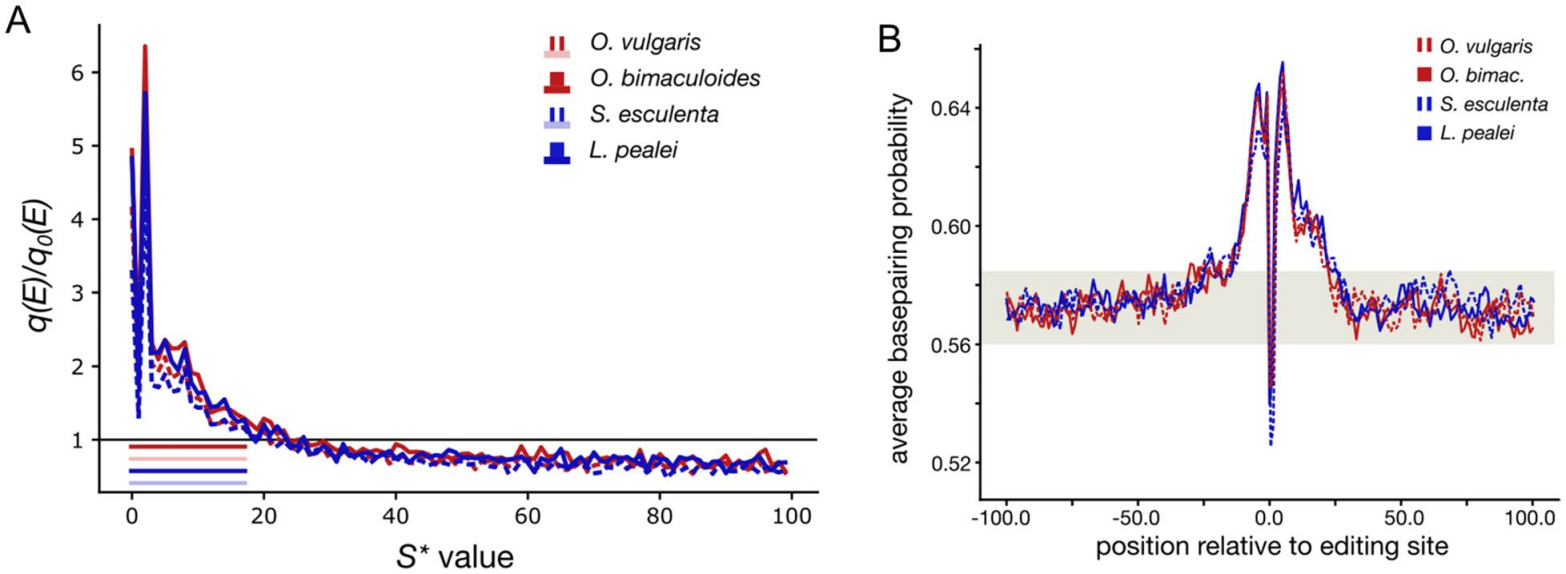
Properties of medium-range clusters of editing sites. (**A**) Deviation of the editing probabilities of adenines located near editing sites (*q*(*E*)) from the respective expected probabilities (*q*_0_(*E*)) as dependent on the S* values. The colored stripes in the lower left corner represent the S* value ranges on which *q*(*E*) are significantly higher than *q*_0_(*E*) (*p* < 0.01, the χ^2^ contingency test, Bonferroni corrected) (**B**) Average base-pairing probabilities in the regions centered at editing sites in four coleoid species. The gray stripe marks the base pairing probability range in regions distant from editing sites (> 200 nt), considered as noise. The values above the noise (the central peak) describe the putative average RNA structure around editing sites; the width of the peak is the average size of the structure. The dip in the middle is caused by generally low base-pairing probabilities of edited adenines.

Previous studies and the observed correlations in the editing state co-occurrence for *S* values larger than 1 (Fig. 2A) hint that A-to-I editing sites may cluster not only in the form of DCs^5,13,32,33,37,47^. Thus, we checked how the distance to the nearest editing site affects the probability of adenine editing (Fig. 4A). We introduce the measure S* defined as the distance between two edited adenines such that no other edited adenine is located between them, and consider the deviation of the observed S* distribution from the expected one (Fig. 4A, Supplementary Fig. S4B). The expected distributions were calculated on randomly generated datasets described above. For all considered coleoid species, the observed and expected S* distributions differ significantly only for windows of up to 18 nucleotides (*p* < 0.01, the χ^2^ test with the Bonferroni correction), thus suggesting a direct dependence of editing events within the 18nt distance.

As noted above, A-to-I editing requires secondary RNA structures to be formed around the edited adenine^2,5,10,28,32,43,44,47^. Hence, the observed clustering of editing sites may be explained by common RNA structures at clustered sites. Thus, we have assessed the average size of a local secondary RNA structure by analyzing average base pairing probabilities of nucleotides around editing sites (Fig. 4B). To control for the accuracy of our predictions of RNA structures around editing sites, we checked for the presence of a well-known effect, where edited adenines tend to form A-C mismatches in RNA double helices more than their non-edited counterparts^5,33,46,47^. Indeed, this effect was substantial (Supplementary Fig. S6) and highly significant (*p* = 5.1 × 10^−34^, Fischer’s exact test).

The average RNA structure size for each coleoid species is determined as the average width of peak in pairing probabilities of nucleotides centered at editing sites; the peak is defined at the region where the average base-pairing probabilities are greater than those of nucleotides distant from editing sites. So defined peaks for all four considered coleoid species fall in the range 32–45 nt, which is consistent with the above estimate of the distance at which an edited adenine influences the probability of editing of a neighboring adenine, which is 2 × 18 nt = 36 nt (Fig. 4A). Thus, the correlated editing of adenines located sufficiently close to each other indeed may be caused by common local secondary RNA structures. Moreover, as there is a higher probability of editing of adenines located in the vicinity of editing sites, editing sites should cluster along the transcript, forming what we call medium-range editing site clusters.

The result about editing sites being less likely involved in secondary RNA structures (Fig. 4B) seemingly contradicts earlier observations that these sites tend to reside within structured regions^5,28,33,43,44,47^. This controversy was resolved by nucleotide-resolution structural analysis of regions around editing sites. For each edited adenine we sampled the nearest non-edited adenine as a control and assessed the site and control base-pairing probabilities (Supplementary Fig. S7). The base-pairing probability of control sites turned out to be larger than that of editing sites, the effect being stronger for sites with large ELs (Supplementary Fig. S7A). Moreover, the energy of the local secondary RNA structure was lower for editing sites compared to that of control ones (Supplementary Fig. S7B), confirming that the RNA structure around editing sites is more stable on average than that at the editing sites themselves. The observed pattern suggests that editing sites generally tend to reside in loops or bulges, i.e. in non-paired regions surrounded by stable helices and are also likely to form A-C mismatches.

### Long-range clusters of editing sites

Earlier studies of coleoid editing sites demonstrated relatively higher sequence conservation in intervals of ± 100–200 nt relative to conserved editing sites^13^ and a correlation between differences in the editing levels at homologous sites and the number of mismatches in the ± 100 nt region^28^. These two consistent estimates indicate that editing at focal sites depends on ± 100–200 nt context, which exceeds the size of medium-range cluster sizes, established above as of 32–45 nt (Fig. 4).

**Figure 5:**
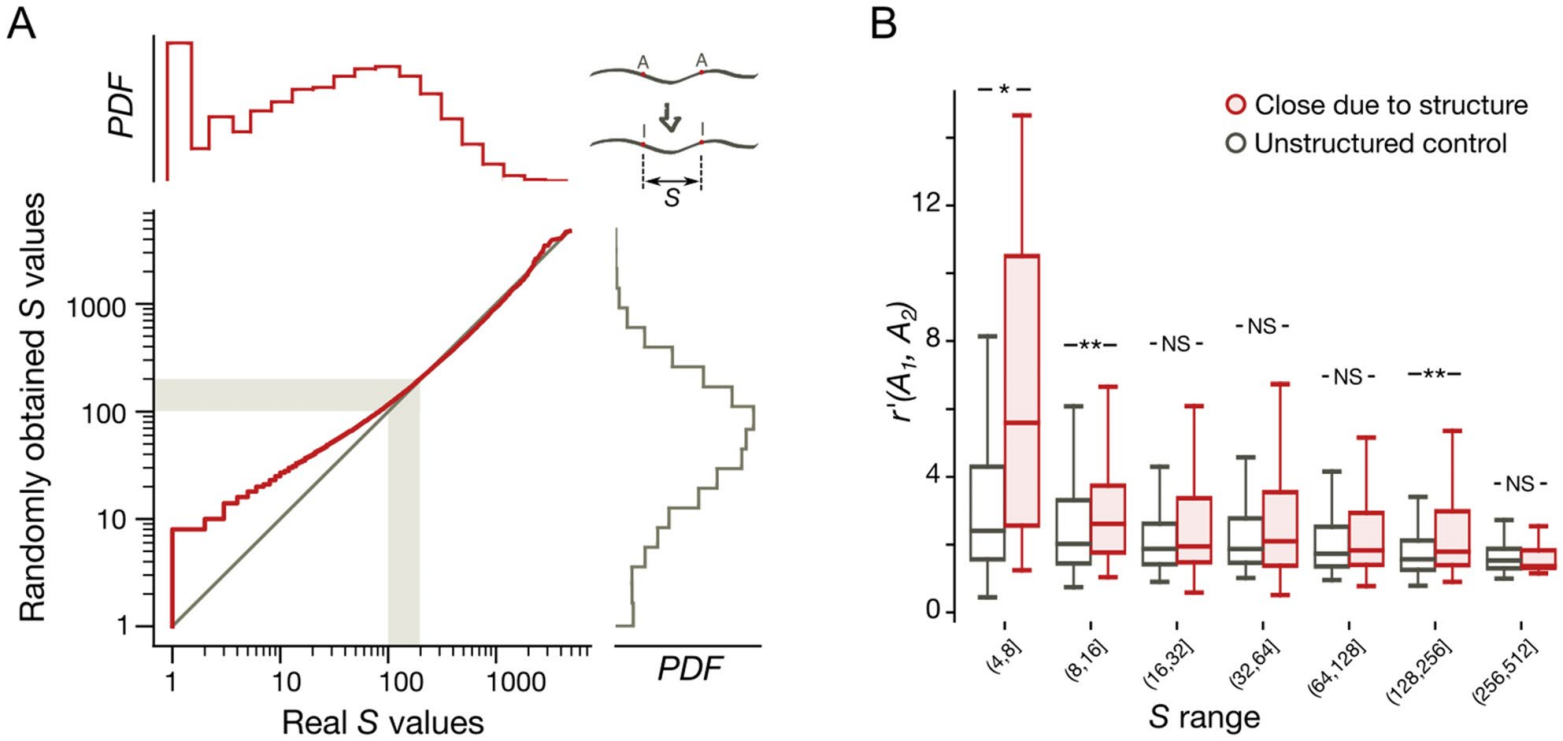
Long-range editing site clusters. (**A**) Distribution of *S* distances for *O. vulgaris*. The real editing site set (red histogram) *vs*. randomly selected adenines (grey histogram), see the text for details. The red line is the plot of dependence between the real and the randomly obtained *S* values in arrays sorted by the distance *S*. The grey diagonal represents the expected dependence form *y* = *x*. Grey stripes represent the boundary of the possible span of regions affecting editing sites^13,28^. PDF—probability density function. (**B**) Distributions of the ${r}^{^{\prime}}\left({A}_{i},{A}_{j}\right)$ values calculated for the structurally close editing sites (red boxes) and for the control site pairs with no predicted secondary RNA structure between the sites in a pair (grey boxes) (see the text for details). Asterisks mark statistical significance of differences of means calculated using the Mann–Whitney U-test with the Bonferroni correction for binning. Two asterisks indicate *p* < 0.01; one asterisk, *p* < 0.05, NS, not significant.

Medium-range clusters have been identified by probability measures. A complementary approach is the comparison of real and expected *S* values, *S* being the distance (in nucleotides) between two edited adenines located in a single transcript, regardless of other possible editing sites between them. As with dense and medium-range clusters, the null models for *S* values were derived from random sets of adenines with the per-transcript number of editing sites preserved and with the tri-nucleotide context preserved (see "Materials and methods"). We have observed that the distribution of distances, *S*, calculated for known coleoid editing site sets is bimodal with a high and distinct peak at 1, reflecting overrepresentation of edited adenines in dense clusters (Fig. 5A, red curve, Supplementary Figs. S4C, S8). Having calculated distances *S* using the randomized set of adenines, we have observed strong and highly significant differences between the real and control *S* distributions (*p* < 2.2 × 10^−308^, the Kolmogorov–Smirnov test, Fig. 5A). At that, the differences are limited to distances *S* smaller than approx. 100–200 nt (Fig. 5, Supplementary Fig. S7), consistent with the earlier observations mentioned above^13,28^, and yields long-range editing site clusters at the scale of 200–400 nt.

To understand the mechanisms yielding long-range clusters, we applied a relaxed definition of RNA structure spanning over a pair of edited adenines. We considered pairs of adenines brought close to each other in space by secondary RNA structure (see Methods). As a control, we considered pairs of sites such that no secondary RNA structure could be identified between them (Fig. 5B, Supplementary Fig. S9). As a measure of co-operativity of editing, we employed the formula: ${r}^{^{\prime}}\left({A}_{i},{A}_{j}\right)= {f}_{i,j}^{II}/{(f}_{i}^{I}{f}_{j}^{I})$, where ${f}_{i,j}^{II}$ is the frequency of co-editing at a pair of sites *i* and *j*, and ${f}_{i}^{I}$ and ${f}_{j}^{I}$ are the individual frequencies of editing at the respective sites. Editing sites brought close by secondary RNA structures were generally more co-operative (*p* = 7.8 × 10^−7^, the Mann–Whitney U-test) than the control sites, with the sites at distances 4–16 nt and 128–256 nt exhibiting significant increase in co-operativity when brought close by secondary RNA structure (*p* < 0.05, the Mann–Whitney U-test with the Bonferroni correction for binning) (Fig. 5B, Supplementary Fig. S9). This result indicates the effects on co-operativity at characteristic long-range cluster sizes to be brought about by secondary RNA structures. These structures could be expected to be rather weak on average, as the structural potentials of nucleotides at distances from editing sites larger than 36 are indistinguishable from noise (Fig. 4B).

### Directionality of editing

**Figure 6:**
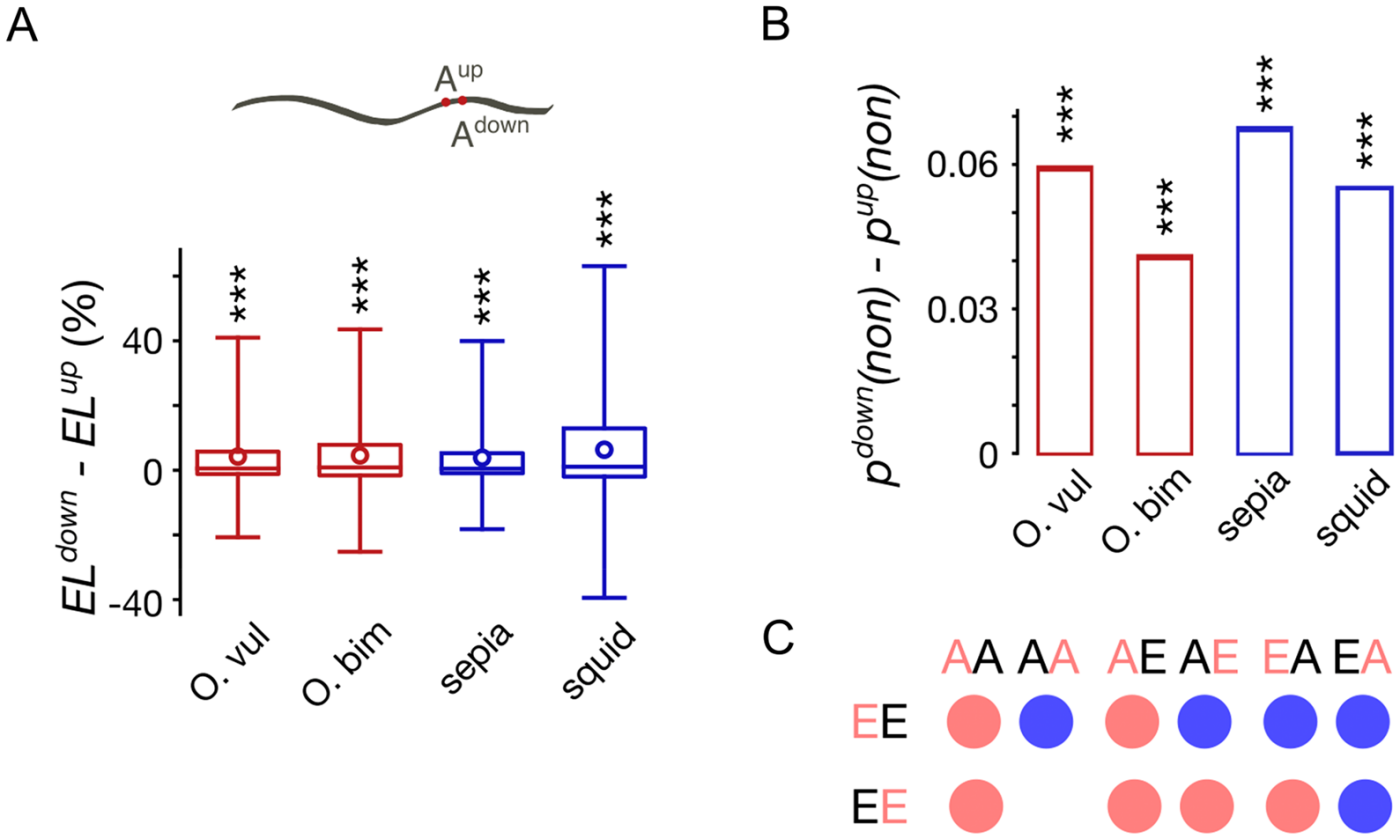
Directionality of dense clusters. (**A**) Distributions of the differences in ELs between down- and upstream editing sites in two-adenine (AA) dense clusters. Three asterisks mark statistical significance of the differences in means (*p* < 0.001, the χ^2^ contingency test). (**B**) Differences between the probabilities of down- and upstream editing sites to be non-synonymous. Three asterisks mark statistical significance of the differences in means (*p* < 0.001, the binomial test). (**C**) Differences in base-pairing probabilities between paired editing sites (EE) and three types of control AA-dinucleotides (see the text for details). Red color of a letter indicates the nucleotide in a dinucleotide, for which base-pairing probabilities are considered. Red and blue circles show significantly lower and higher base-pairing probabilities for the EE dinucleotide compared to the respective control (the Wilcoxon test *p* < 0.05, Bonferroni corrected).

As noted above, the strongest association in terms of EL or the co-occurrence of edited states is observed for adjacent editing sites (*S* = 1) (Fig. 2A,B), with the two-adenine (AA) clusters comprising the vast majority of dense clusters (Fig. 3A). The observed effects may be due to co-operativity of editing, so that, if an adenine is edited, this would enhance the editing context for an adjacent adenine. The editing context is asymmetric (Fig. 1B), hence we expect probabilities of editing of adenines located immediately up- and downstream from an editing site to differ. Moreover, the contextual features of editing sites were hypothesized to yield AI rather than IA as the preferred intermediate to the II dinucleotide in paired editing events, and consequently more AI-reads were observed in paired editing sites of coleoids^37^. Indeed, the ELs at downstream sites are on average 4–6% higher than those of the upstream ones (*p* < 1.5 × 10^−80^, the Wilcoxon signed-rank test) and this result does not depend on the position of the AA-cluster relative to the reading frame of the coding sequence (Fig. 6A, Supplementary Fig. S10). Thus, the dynamics of editing of AA-clusters manifests as general differences in ELs at the up- and downstream adenines in DCs.

Re-coding (non-synonymous) A-to-I editing in coleoids might be beneficial, as it diversifies the proteome and, consequently, allows for appropriate phenotypic and evolutionary responses to novel environments^13,14,25,28,29^. In line with this reasoning coupled with the observation that downstream sites in AA-clusters were more prone to editing, we compared the fraction of sites with non-synonymous A-to-G substitutions among up- and downstream adenines in AA-clusters (Fig. 6B), where both adenines were edited, with the corresponding fractions in AA dinucleotides, where both adenines were not edited. The probabilities of the downstream sites to be re-coding was higher than those for the upstream sites (*p* < 3 × 10^−6^, the binomial test) even accounting for differences in the probabilities of editing in AA dinucleotides.

The differences between ELs and the fractions of re-coding sites of up- and downstream paired edited adenines may be also explained by features of the local secondary RNA structure required for the ADAR action^2^. We assessed the latter explanation by calculating the probabilities of each nucleotide to be involved in secondary RNA structures, which we refer to as the base-pairing probabilities (see Methods). For each paired editing site (EE-site), we considered the base-pairing probabilities of up- and downstream editing sites separately. As controls, we considered three sets of AA dinucleotides located within ± 20 nt windows around EE-sites: (1) pairs of non-edited adenines (AA-sites), (2) downstream-edited and upstream-unedited adenines (AE-sites), and (3) upstream-edited and downstream-unedited adenines (EA-sites). If none of the controls could be obtained for an EE-site, it was not considered further (Fig. 6C, Supplementary Tables S3, S4). As in the case with EE-sites, we considered base-pairing probabilities in control dinucleotides separately for up- and downstream nucleotides.

Firstly, we observed the base-pairing probabilities of downstream adenines in EE-sites to be significantly lower than those of upstream adenines (Wilcoxon *p* < 2.6 × 10^−39^). The dependency of base-pairing probabilities on the adenine position in a dinucleotide extends to the comparison of base-pairing probabilities of EE-sites with those of control dinucleotides (Fig. 6C, Supplementary Tables S3, S4), where the downstream adenine seems to be generally less structured than the upstream adenine. Additionally, positions of editing sites in the control sets largely and consistently affect the results: AE-sites are generally more structured than EA-sites (Fig. 6C). Thus, the downstream adenines in EE-sites are edited more frequently, are more likely to be re-coding if edited, and are less likely to be involved in secondary RNA structure.

These results suggest that editing at downstream sites is the primary event in DC editing, which may be followed by editing at upstream sites. To check this hypothesis, we reconstructed the temporal sequences of editing events in *O. vulgaris* transcriptome using an approach similar to the one of ref.^45^ (see Supplementary Methods). For coupled editing sites, we observed a significant tendency for the downstream sites to be edited prior to the upstream ones (Wilcoxon *p* = 3.8 × 10^−35^). One possible explanation for that would be a general tendency of ADARs to edit firstly down- and then upstream sites located nearby. To check it, we considered paths of editing events, where the pairs of editing sites are separated by more than one nucleotide (*S* > 1). As in the case with coupled editing sites, we observed a significantly larger number of paths where downstream adenines were edited prior to the upstream ones (Wilcoxon *p* = 1.4 × 10^−96^). Thus, at least to some extent, the directionality in DC editing is explained by the general directionality of editing. However, this result does not rule out an alternative possibility that changes in the local context of upstream sites introduced by editing at downstream sites induce editing at upstream sites, as suggested by the established editing site context, where the preferential downstream nucleotide for an edited adenine is guanine (Fig. 1B).

## Discussion

### Cooperativity of RNA editing

A-to-I editing sites in coleoid genomes tend to cluster. The strength of correlations in the editing state co-occurrence clearly depends on the distance between the sites. One explanation is provided by the common secondary RNA structure formed around closely located editing sites. However, the common RNA structures do not explain the inosine co-occurrence observed here (Fig. 2A) and in other studies^5,13,33,37,43,44,47^. Indeed, suppose an adenine is edited due to the local RNA structural features. The local structure would generally enhance the probability of editing of adjacent adenines^36^, however, editing at an adjacent site would not depend on the editing at the considered site, unless the RNA structure has changed due to the first act of editing. Thus, no correlations would be observed. This prompts for a dynamic explanation based on changes of editing probabilities near the focal site introduced by editing at that site. We consider the following two scenarios: (1) ADAR enzymatic action at adjacent editing sites is co-operative, manifesting as simultaneous adenine editing dependent on the linear distance between the edited adenines and (2) inosine produced by editing at one site stabilizes the existing local secondary RNA structure or even causes RNA to fold in a different manner, hence enhancing the probabilities of editing at nearby adenines.

The former explanation presumes that ADAR enzymes can edit multiple sites in a series of enzymatic acts, this ability being dependent on inter-site distances. This is indirectly supported by the fact that different ADAR subunits show enzymatic cooperativity for substrate binding^49^. Similar effects are observed e.g. in the case of co-operative phosphorylation of adjacent amino acids in proteins, where clusters of phosphorylated residues form due to the enzymatic features of phosphatases^50–52^. That, however, does not explain the prevailing editing state co-occurrence in the adjacent adenines, as two ADAR subunits may not physically edit two consecutive adenines simultaneously^53^. But there may be slippage of the ADAR RNA-binding domain on the RNA sequence, resulting in editing of the adjacent adenine.

In the RNA-centered model, the seeming co-operativity of A-to-I editing of adjacent sites is attributed to the reinforcement of the local secondary RNA structure, which would increase the probabilities of editing at adjacent or closely positioned adenines. Inosines form base-pairs with cytosines, the I-C base pair being isosteric to, but slightly less stable than the G–C pair^54^. Together with our observation about edited adenines forming frequent A–C mismatches in the local structure^5,33,46,47^ (Supplementary Fig. S6), this points to a possibility that editing at a focal site changes the local RNA structure pattern, reinforcing the propensity towards stronger secondary structure, and hence promotes editing at adenines in the vicinity. We could not test this explanation computationally due to insufficient data on structural features of inosines^54^.

The editing of coupled adenines seems to be consistent with the RNA-centered model and follow the scenario involving two factors: dynamics of the sequence context^37^ and dynamics of the local RNA structure. First, the downstream adenine in a pair is edited due to the upstream adenine being the preferred context (Fig. 1B) and due to the larger accessibility to ADAR as a non-structured element in a secondary structure (Fig. 6C). As a result, the context of the upstream site changes to upstream I instead of A. At that, guanine, an analogue to inosine is the preferred downstream context for editing (Fig. 1B). Along with that, the local RNA structure may be reinforced, specifically due to inosine pairing with cytosine^5,33,46,47^. These two factors may pave the way for editing of the upstream adenine. This scenario implies editing of the upstream adenine to be largely a mechanistic consequence of editing of the downstream adenine. While this scheme may not be true in all cases, we observe downstream adenines to be more frequently re-coding and hence possibly more frequently selected upon than their upstream counterparts. Thus, in a large number of cases, editing of upstream adenines may indeed be guided by contextual and structural changes induced by the editing at downstream sites. However, this does not explain the phenomenon of directional editing in non-DC clusters, which may be a consequence of specific ADAR activities.

### The range of influence of editing sites

Previous studies have established the linear lengths of RNA structures associated with A-to-I mRNA editing to be of various sizes ranging from rather short structures^30^ to complex formations spanning over large fragments of the transcript^5^. In coleoid coding sequences, conserved regions around conserved editing sites span on average 100–200 nt in each direction^13^. Accordingly, clustering of edited adenines obtained from the *S* value analysis and the analysis of structurally close edited adenines is observed at up 100–200 nt and up to 256 nt, respectively (Fig. 2A). However, the analysis of adenine editing probabilities in the vicinity of edited sites (Supplementary Fig. S2) and the analysis of base-pairing probabilities in the regions around edited adenines (Fig. 4C) have yielded different and consistent estimates of 36 nt and 32–45 nt, respectively. This indicates a hierarchy in the cluster structure, with relatively large, diffuse clusters yielded possibly by weak secondary RNA structures associated with editing sites, which span up to 256 nt (Fig. 6). Smaller, however more stable structures spanning up to 45 nt yield the intermediate level of clustering (Fig. 5). Finally, the local features of RNA structure, e.g. loops, mismatches or bulges, confer the strongest association in terms of editing, which manifests as clusters of adjacent edited adenines (Figs. 2, 4C).

One important limitation of our and other similar studies is that the existence of introns is largely ignored. Indeed, editing involves unspliced transcripts, whereas one cannot infer the editing state of intronic adenines from the sequenced mRNA data. However, according to the annotation^55^, an average adenine in the transcriptome is expected to be located in a 1467 nt exon, which is at least several fold larger than the distances considered here; hence, our observations should not be affected by the exon–intron structure to a considerable extent. Indeed, the analysis of relaxed long-range structures that considers the longest distances (Fig. 5B) yields the same results when only exons are considered instead of transcripts (Supplementary Fig. S9). Nonetheless, the lack of data on the exon–intron structures in coleoids may explain an apparent discrepancy between a typical cluster size and the observations of editing eQTLs^35^ and RNA secondary structures^5^ spanning thousands of nucleotides. A simpler alternative, of course, is that large-scale statistical studies may not detect rare and long-range effects.

## Materials and methods

### Data

We used previously published transcriptomes^13^ of *O. vulgaris*, *O. bimaculoides*, *S. esculenta*, and *L. pealei* along with the publicly available coleoid editing sites data^13^. The corresponding transcriptomic read data, summarized in Supplementary Table S1, were downloaded from the SRA database. For each species, corresponding SRA files were pooled. For the analysis of exons, we used the publicly available genomic sequences and annotation of *O. bimaculoides*^55^.

### Data analysis

Reads were mapped with the bowtie2 package^56^. RNA structural annotation was performed with the RNAsurface program^57^ and the plfold algoritm of the Vienna package^58^. For Fig. 5B, every such pair was assigned to one of the three groups: “close due to structure”, “distant, unstructured”, or “intermediate” based on the linear distance and the existence of secondary structure between the sites; for details see Supplementary Methods.

### Calculation of S values

*S* values were calculated as nucleotide distances between edited adenines on transcripts. Along with *S* values calculated for actual editing sites, we calculated *S* values for randomly selected adenines accounting for possible biases, see Supplementary Methods. The S* values were calculated as nucleotide distances between subsequent edited adenines, i.e. for pairs of editing sites with no edited adenines between them.

### Statistics

The tendency of editing states to co-occur on the transcripts and correlations between the editing levels at pairs of sites were assessed with the Pearson’s correlation^59^. The confidence intervals and the significance of each correlation coefficient were inferred using the t-test with the Bonferroni correction^60^ for multiple testing. The distributions of *S* values were compared using the two-sample Kolmogorov–Smirnov test^61^. Editing levels, the distributions of correlation coefficients, and the distributions of structural potential Z-scores were compared with the Mann–Whitney U test^62^. The editing levels at upstream and downstream editing sites were compared with the Wilcoxon’s signed-rank test^63^.

The grouping of *S* values with respect to the differences in correlations between edited states on transcripts was performed using the Mann–Whitney U test: for each pair of correlation arrays corresponding to different *S* value ranges, the Mann–Whitney statistic was calculated, and groups of *S* value ranges were further defined as the groups of sequential ranges differing insignificantly from each other.

## Supplementary Information

Supplementary Information.

## Data Availability

All data analyses were performed in Python 3.7. Scripts and data analysis protocols are available online at https://github.com/mikemoldovan/coleoidRNAediting2.

## References

[1] Bass BL, Weintraub H (1988). An unwinding activity that covalently modifies its double-stranded RNA substrate. Cell.

[2] Reenan RA (2005). Molecular determinants and guided evolution of species-specific RNA editing. Nature.

[3] Yang Y (2008). A-to-I RNA editing alters less-conserved residues of highly conserved coding regions: Implications for dual functions in evolution. RNA.

[4] Ensterö M, Daniel C, Wahlstedt H, Major F, Öhman M (2009). Recognition and coupling of A-to-I edited sites are determined by the tertiary structure of the RNA. Nucleic Acids Res..

[5] Morse DP, Aruscavage PJ, Bass BL (2002). RNA hairpins in noncoding regions of human brain and *Caenorhabditis elegans* mRNA are edited by adenosine deaminases that act on RNA. Proc. Natl. Acad. Sci..

[6] Xu G, Zhang J (2014). Human coding RNA editing is generally nonadaptive. Proc. Natl. Acad. Sci. USA.

[7] Wahba AJ (1963). Synthetic polynucleotides and the amino acid code, VIII. Proc. Natl. Acad. Sci..

[8] Sommer B, Köhler M, Sprengel R, Seeburg PH (1991). RNA editing in brain controls a determinant of ion flow in glutamate-gated channels. Cell.

[9] Nishikura K (2006). Editor meets silencer: Crosstalk between RNA editing and RNA interference. Nat. Rev. Mol. Cell Biol..

[10] Nishikura K (2010). Functions and regulation of RNA editing by ADAR deaminases. Annu. Rev. Biochem..

[11] Nishikura K (2015). A-to-I editing of coding and non-coding RNAs by ADARs. Nat. Rev. Mol. Cell Biol..

[12] Alon S (2012). Systematic identification of edited microRNAs in the human brain. Genome Res..

[13] Liscovitch-Brauer N (2017). Trade-off between transcriptome plasticity and genome evolution in cephalopods. Cell.

[14] Eisenberg E, Levanon EY (2018). A-to-I RNA editing—Immune protector and transcriptome diversifier. Nat. Rev. Genet..

[15] Garrett S, Rosenthal JJC (2012). RNA editing underlies temperature adaptation in K+ channels from polar octopuses. Science.

[16] Feldmeyer D (1999). Neurological dysfunctions in mice expressing different levels of the Q/R site-unedited AMPAR subunit GluR-B. Nat. Neurosci..

[17] Brusa R (1995). Early-onset epilepsy and postnatal lethality associated with an editing-deficient GluR-B allele in mice. Science.

[18] Maas S, Kawahara Y, Tamburro KM, Nishikura K (2006). A-to-I RNA editing and human disease. RNA Biol..

[19] Yablonovitch AL, Deng P, Jacobson D, Li JB (2017). The evolution and adaptation of A-to-I RNA editing. PLoS Genet..

[20] Ramaswami G (2012). Accurate identification of human Alu and non-Alu RNA editing sites. Nat. Methods.

[21] Kim DDY (2004). Widespread RNA editing of embedded Alu elements in the human transcriptome. Genome Res..

[22] Pinto Y, Cohen HY, Levanon EY (2014). Mammalian conserved ADAR targets comprise only a small fragment of the human editosome. Genome Biol..

[23] Yu Y (2016). The landscape of A-to-I RNA editome is shaped by both positive and purifying selection. PLoS Genet.

[24] Alon S (2015). The majority of transcripts in the squid nervous system are extensively recoded by A-to-I RNA editing. Elife.

[25] Shoshan Y, Liscovitch-Brauer N, Rosenthal JJC, Eisenberg E (2021). Adaptive proteome diversification by nonsynonymous A-to-I RNA editing in coleoid cephalopods. Mol. Biol. Evol..

[26] Chen L (2013). Characterization and comparison of human nuclear and cytosolic editomes. Proc. Natl. Acad. Sci..

[27] Jiang D, Zhang J (2019). The preponderance of nonsynonymous A-to-I RNA editing in coleoids is nonadaptive. Nat. Commun..

[28] Moldovan M, Chervontseva Z, Bazykin G, Gelfand MS (2020). Adaptive evolution at mRNA editing sites in soft-bodied cephalopods. PeerJ.

[29] Popitsch N (2020). A-to-I RNA editing uncovers hidden signals of adaptive genome evolution in animals. Genome Biol. Evol..

[30] Savva YA, Rieder LE, Reenan RA (2012). The ADAR protein family. Genome Biol..

[31] Eggington JM, Greene T, Bass BL (2011). Predicting sites of ADAR editing in double-stranded RNA. Nat. Commun..

[32] Nishikura K (1991). Substrate specificity of the dsRNA unwinding/modifying activity. EMBO J..

[33] Morse DP, Bass BL (1999). Long RNA hairpins that contain inosine are present in *Caenorhabditis elegans* poly(A)+ RNA. Proc. Natl. Acad. Sci..

[34] Paz-Yaacov N, Levanon EY, Nevo E, Kinar Y, Harmelin A, Jacob-Hirsch J, Amariglio N, Eisenberg E, Rechavi G (2010). Adenosine-to-inosine RNA editing shapes transcriptome diversity in primates. Proc. Natl. Acad. Sci..

[35] Kurmangaliyev YZ, Ali S, Nuzhdin SV (2016). Genetic determinants of RNA editing levels of ADAR targets in *Drosophila melanogaster*. G3 Genes Genomes Genet..

[36] Gommans WM, Mullen SP, Maas S (2009). RNA editing: A driving force for adaptive evolution?. BioEssays.

[37] Duan Y (2017). Linkage of A-to-I RNA editing in metazoans and the impact on genome evolution. Mol. Biol. Evol..

[38] Polson AG, Bass BL (1994). Preferential selection of adenosines for modification by double-stranded RNA adenosine deaminase. EMBO J..

[39] Zhang Z, Carmichael GG (2001). The fate of dsRNA in the nucleus. Cell.

[40] Prasanth KV (2005). Regulating gene expression through RNA nuclear retention. Cell.

[41] Li Q (2014). Caste-specific RNA editomes in the leaf-cutting ant *Acromyrmex echinatior*. Nat Commun.

[42] Zhang R, Deng P, Jacobson D, Li JB (2017). Evolutionary analysis reveals regulatory and functional landscape of coding and non-coding RNA editing. PLoS Genet..

[43] Levanon EY, Eisenberg E (2014). Does RNA editing compensate for Alu invasion of the primate genome?. BioEssays.

[44] Athanasiadis A, Rich A, Maas S (2004). Widespread A-to-I RNA editing of Alu-containing mRNAs in the human transcriptome. PLoS Biol..

[45] Barak M, Levanon EY, Eisenberg E, Paz N, Rechavi G, Church GM, Mehr R (2009). Evidence for large diversity in the human transcriptome created by Alu RNA editing. Nucleic Acids Res..

[46] Wong SK, Sato S, Lazinski DW (2001). Substrate recognition by ADAR1 and ADAR2. RNA.

[47] Kallman AM (2003). ADAR2 A->I editing: Site selectivity and editing efficiency are separate events. Nucleic Acids Res..

[48] Hedges SB, Dudley J, Kumar S (2006). TimeTree: A public knowledge-base of divergence times among organisms. Bioinformatics.

[49] Valente L, Nishikura K (2007). RNA binding-independent dimerization of adenosine deaminases acting on RNA and dominant negative effects of nonfunctional subunits on dimer functions. J. Biol. Chem..

[50] Al-Khouri AM, Ma Y, Togo SH, Williams S, Mustelin T (2005). Cooperative phosphorylation of the tumor suppressor phosphatase and tensin homologue (PTEN) by casein kinases and glycogen synthase kinase 3β. J. Biol. Chem..

[51] Schweiger R, Linial M (2010). Cooperativity within proximal phosphorylation sites is revealed from large-scale proteomics data. Biol. Direct.

[52] Moldovan M, Gelfand MS (2020). Phospho-islands and the evolution of phosphorylated amino acids in mammals. PeerJ.

[53] Stefl R (2010). The solution structure of the ADAR2 dsRBM-RNA complex reveals a sequence-specific readout of the minor groove. Cell.

[54] Wright DJ, Force CR, Znosko BM (2018). Stability of RNA duplexes containing inosine·cytosine pairs. Nucleic Acids Res..

[55] Albertin CB, Simakov O, Mitros T, Wang ZY, Pungor JR, Edsinger-Gonzales E, Brenner S, Ragsdale CW, Rokhsar DS (2015). The octopus genome and the evolution of cephalopod neural and morphological novelties. Nature.

[56] Langmead B, Salzberg SL (2012). Fast gapped-read alignment with Bowtie 2. Nat. Methods.

[57] Soldatov RA, Vinogradova SV, Mironov AA (2013). RNASurface: Fast and accurate detection of locally optimal potentially structured RNA segments. Bioinformatics.

[58] Lorenz R (2011). ViennaRNA package 2.0. Algorithms Mol. Biol..

[59] Pearson K (1895). Notes on regression and inheritance in the case of two parents. Proc. R. Soc. Lond..

[60] Bonferroni CE (1936). Teoria statistica delle classi e calcolo delle probabilità.

[61] Kolmogorov AN (1933). Sulla determinazione empirica di una legge di distribuzione.

[62] Mann HB, Whitney DR (1947). On a test of whether one of two random variables is stochastically larger than the other. Ann. Math. Stat..

[63] Wilcoxon F (1945). Individual comparisons by ranking methods. Biom. Bull..

[64] Nguyen L-T, Schmidt HA, von Haeseler A, Minh BQ (2014). IQ-TREE: A fast and effective stochastic algorithm for estimating maximum-likelihood phylogenies. Mol. Biol. Evol..

[65] Sagulenko P, Puller V, Neher RA (2018). TreeTime: Maximum-likelihood phylodynamic analysis. In Virus Evol..

