# Supplementary material for: A hierarchy in clusters of cephalopod mRNA editing sites
Source: Sci Rep. 2022 Mar 2;12:3447. doi: 10.1038/s41598-022-07460-5 (PMC8891338; doi:10.1038/s41598-022-07460-5)
Supplement: Supplementary file 1 — Supplementary Information. [file 41598_2022_7460_MOESM1_ESM.docx]

**Supplementary materials for
“A hierarchy in clusters of cephalopod mRNA editing sites”**

**Supplementary Methods**

**Calculation of *S* values**. *S* values were calculated as nucleotide distances between edited adenines on transcripts. Along with *S* values calculated for actual editing sites, we calculated *S* values for randomly selected adenines. To eliminate the biases caused by factors such as the higher accuracy of editing sites prediction in highly expressed transcripts or the general tendency of some transcripts to be edited more frequently than others, we have randomly selected in each transcript the number of adenines equal to the number of editing sites it contains. *S** values were calculated as nucleotide distances between subsequent edited adenines, i.e., for pairs of editing sites with no edited adenines between them.

**Control sets of adenines**. As a uniform control, we constructed a random set of adenines as follows: in each transcript we selected the number of random adenines exactly equal to the number of editing sites in this transcript. By using this control instead of a simpler uniform expectation, we address the issue of non-uniformity of adenine occurrence along the transcripts and, in particular, possible overrepresentation of AA dinucleotides.

In addition, we constructed a second set of random adenines such that the three-nucleotide context of randomly chosen adenine set matched that of editing sites (Fig. 1B). Thus, we addressed the non-uniformity of distribution of adenines in specific contexts along the transcripts. The procedure was as follows: for each transcript and for each editing site, we considered its 3-nucleotide context. Next, we selected a random adenine in the same transcript in the same context. This procedure ensures that the number of sites in the control set is exactly the same as the number of editing sites and the contexts of the control set exactly matches that of the edited adenine dataset.

**Editing state co-occurence**. To infer the tendency of edited states to co-occur in transcripts, we have calculated the Pearson correlation [59] of edited state occurrences for all pairs of edited adenines located within the window with the radius equal to the read length.

The reads were mapped onto the transcripts with the bowtie2 package [56] using the –sensitive-local alignment mode. We filtered out read alignments that did not contain regions of continuous read mappings larger than half the read length. The resulting alignment files were further processed with a set of ad hoc scripts and the numbers of editing state occurrences for each considered site pair were calculated.

**Variance due to editing**. To estimate the variance in transcriptomes conferred by editing, at each position, we formally assigned values 0 and 1 respectively to edited and non-edited reads mapping to this position. Thus, the variance at a position is simply *EL*(1*–EL*), where *EL* stands for the editing level at the considered position. Alternatively, this can be written as $f_{i}^{A}f_{i}^{I}$, as in the main text. The additive variance component is thus the sum of $f_{i}^{A}f_{i}^{I}$ over all edited adenines in the transcriptome. The net variance is calculated as the sum of all variances and covariances in the form of $f_{i,j}^{AA}f_{i,j}^{II}- f_{i,j}^{AI}f_{i,j}^{IA}$ (see text for details). Additionally, all between-site covariances considered in this analysis had to be significant (*p* < 0.05, t-test with FDR correction for multiple testing), otherwise they were formally set to zero.

**RNA structural annotations**. To estimate the propensity of sequences to form RNA secondary structure, we have calculated the structural potential Z-scores for each nucleotide using the RNASurface program [57]. *Z-score* is defined as *Z* = $(E- \mu)/\sigma$ with *E*, $\mu$ and $\sigma$ being the minimal free energy of a considered cequence, mean and standard deviation of the free energy distribution of shuffled sequences with preserved length and average dinucleotide composition, respectively. RNASurface was run with the maximal and minimal sliding window length set to 350 and 20, respectively. For each position, *Z-score* was inferred as the minimal *Z-score* of all windows containing it.

The base pairing probabilities were calculated with the plfold algorithm of the Vienna package [58] with –W and –L parameters set to sequence lengths and –cutoff parameter set to 0.0.

For the analysis of editing sites brought close by secondary RNA structures, all possible pairs of editing sites for each transcript were considered. For Fig. 6b, every such pair was assigned to one of the three groups: “close due to structure”, “distant, unstructured”, or “intermediate”. Two editing sites were considered close due to the structure if the distance between them in the structure was less than the distance between them taken by sequence, and, additionally, the distance in structure was less than eight nucleotides. The pair of sites were considered distant if the distance between the sites in the structure was equal to the distance by the sequence or was more than 40 nucleotides. The distance in the structure for the pair of sites was computed as the minimal distance between them in the graph of the transcript with all the potentially paired base pairs and all nucleotides adjacent in the sequence connected by edges. The graph was obtained using the Vienna RNAplfold program with the 0.8 cut-off for the pairing probability [58].

**Structural mismatch annotations.** The tendency of edited adenines to be opposite cytosines in RNA structures was estimated as follows. Given probabilities for every two nucleotides to be paired in a structure, we selected all adenines sandwiched between two base pairs with pairing probability higher than 0.7 and having one-nucleotide symmetrical bubble in between. In total, we obtained sets of 455 and 4298 edited and non-edited, respectively, adenines in such structures. Then we compared the distribution of such mismatch partners for the edited adenines and for the nonedited ones (Supplementary Figure S6). The probability of pairing was computed using RNAplfold [58] for all exon sequences as they would have been prior to editing events.

**Order of editing events.** In each 50 nt window in the read alignment on the transcriptome of *O. vulgaris*, we looked for at least 5 editing sites covered by at least 20 reads. Further, we required each transcript state, defined as the editing pattern in the read, to be supported by at least 5 reads, and the number of states to be at least 4 if one of the states represented the all-A (not edited) state and at least 3 otherwise. If the alignment lacked an all-A state, this state was added artificially as an outgroup. Next, trees were built upon the selected A-I sites treated as polymorphisms by the maximum-likelihood algorithm implemented in the IQ-TREE package [64]. The All-A states were used as outgroups. Quasi-ancestral states representing editing intermediates were reconstructed with the TreeTime package [65]. To control for the quality of the tree reconstruction, we additionally filtered out 13% of the constructed trees where I-to-A substitutions were identified, as the A-to-I editing is irreversible.

An editing path is defined as the sequence of editing events that occur when travelling from the root (all-A sequence) to the leaves of the tree. Additionally, the weight of each A-to-I substitution event was defined as the number of reads representing descendants of this event. The weight of a path was then defined as the weight of the corresponding terminal leaf, which corresponded to the number of reads in one specific editing state. In other words, the weight of an editing path is the minimal weight of an event in this path and represents the number of edited reads that emerged as a consequence of a specific path of editing events. To obtain the resulting ensemble of editing paths, we pooled the constructed paths with corresponding weights. The resulting ensemble consisted of 1529 different editing paths with the total weight of 51326.

**Supplementary Figures**


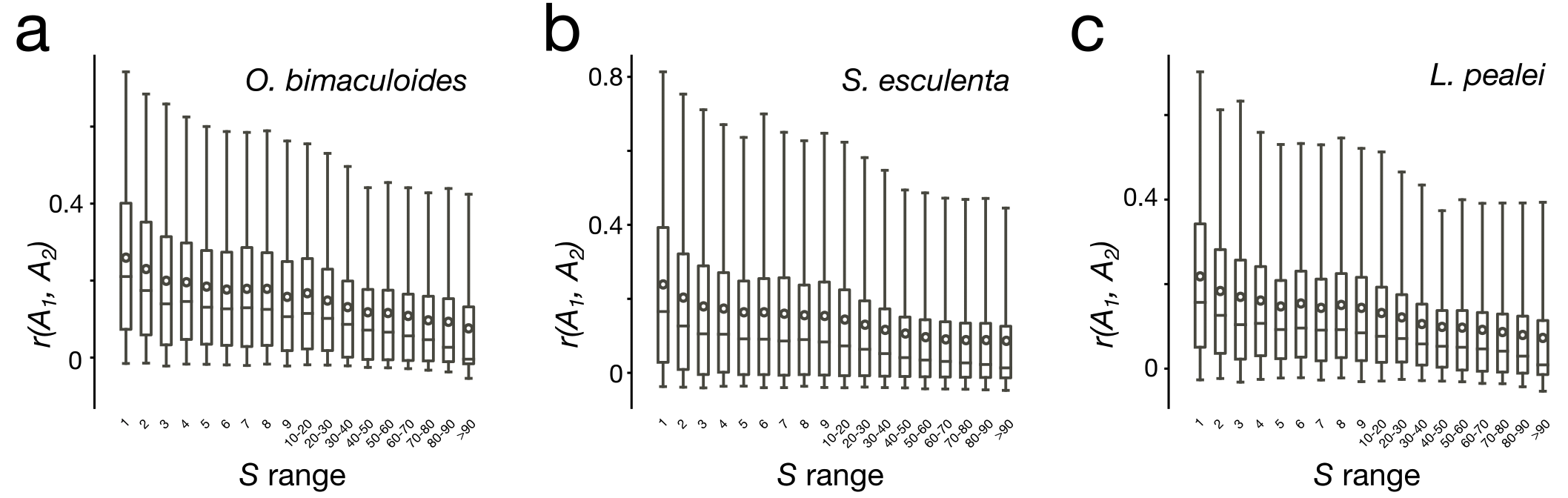


**Supplementary Figure S1 | The distributions of correlation coefficients of coleoid editing at two sites with respect to the distances between sites.** Boxes represent the quartile borders; red circles represent the means; the grey lines indicate the 95% two-sided confidence intervals of the distributions. **(a)** *O. bimaculoides* **(b)** *S. esculenta* **(c)** *L. pealei*. Notation as in Fig. 2a.


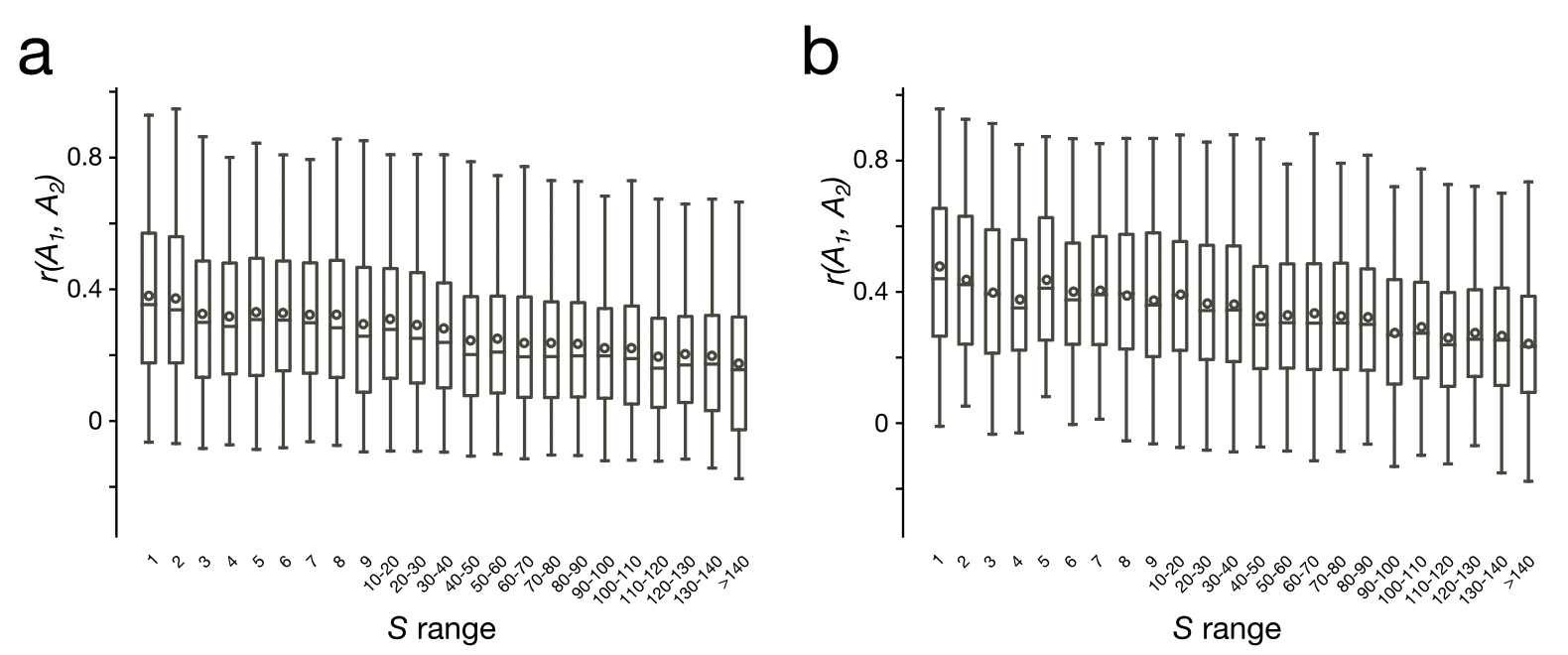


**Supplementary Figure S2 | The distributions of correlation coefficients of *O. vulgaris* editing at two sites with respect to the distances between sites for different minimal editing level threshold values.** Boxes represent the quartile borders; red circles represent the means; and the grey lines indicate the 95% two-sided confidence intervals of the distributions. **(a)** Threshold set to 5% **(b)** Threshold set 10%. Notation as in Fig. 2a, Suppl. Fig. S1.


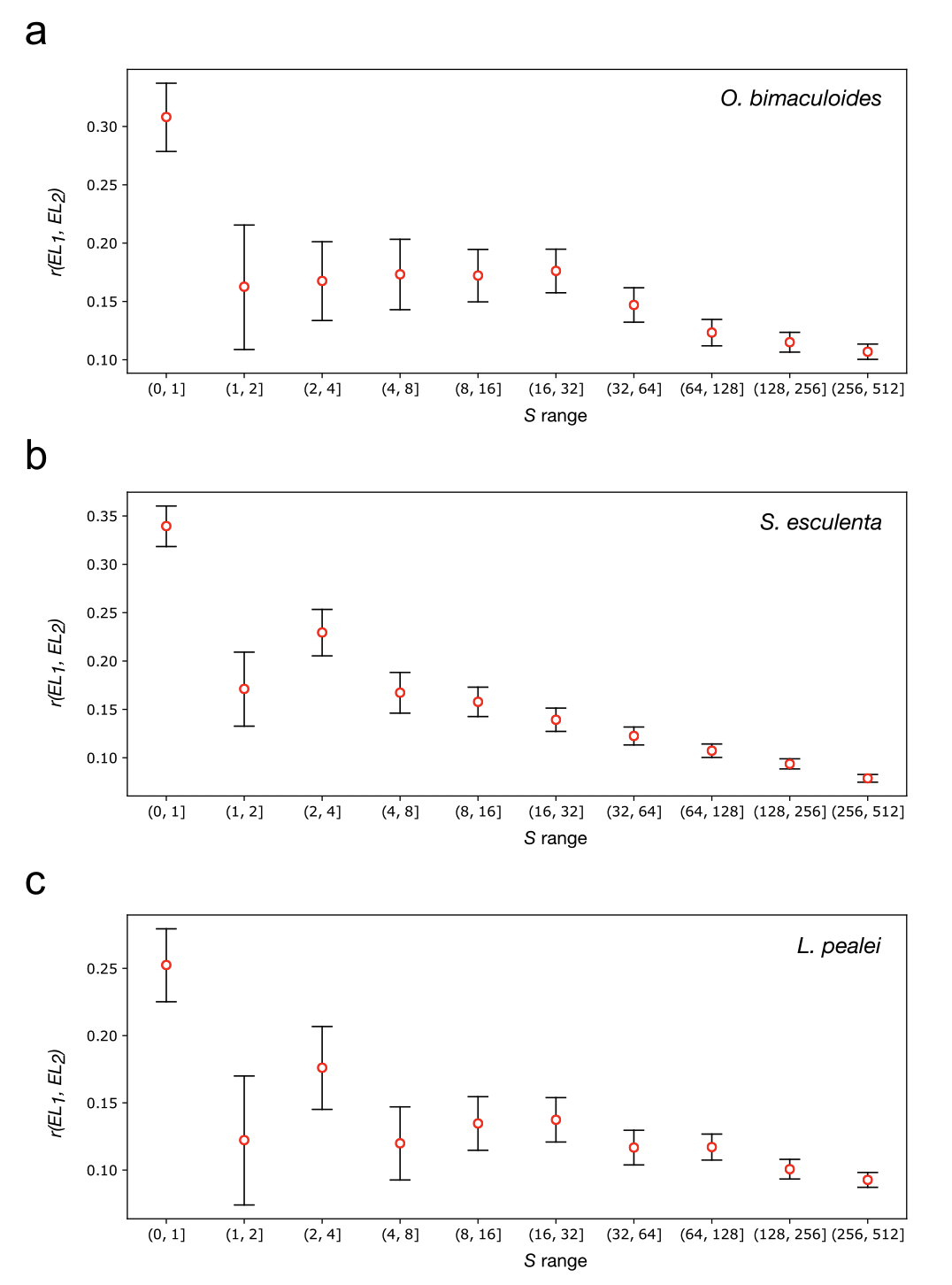


**Supplementary Figure S3 | The dependence of the correlations of ELs on the *S* values for the considered coleoid A-to-I editing site datasets**. The red circles mark the values of the correlation coefficients, and the grey lines represent the Bonferroni corrected 95% two-sided confidence intervals obtained from the *t*-distribution. **(a)** *O. bimaculoides* **(b)** *S. esculenta* **(c)** *L. pealei*. Notation as in Fig. 2b.


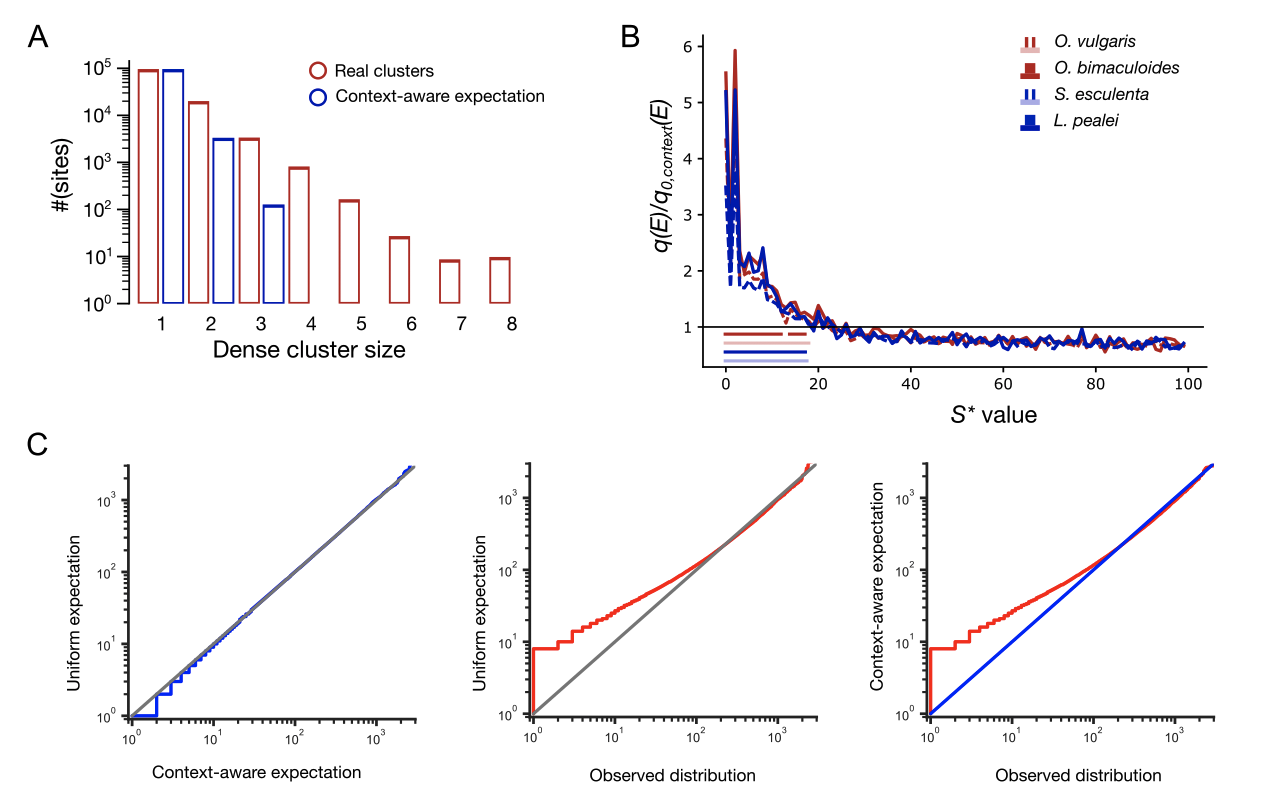


**Supplementary Figure S4 | Results obtained with the context-aware expectation of the distribution of edited adenine. (A)** Histogram of dense cluster sizes (nt) for the real *O. vulgaris* editing site dataset (red) and the matching random context-aware dataset (blue). **(B).** Deviation of the editing probabilities of adenines located near editing sites (*q*(*E*)) from the respective expected probabilities (*q*_0_(*E*)) as dependent on the *S** values. Notation as in Fig. 5A. **(C)** Comparisons of all three distributions.


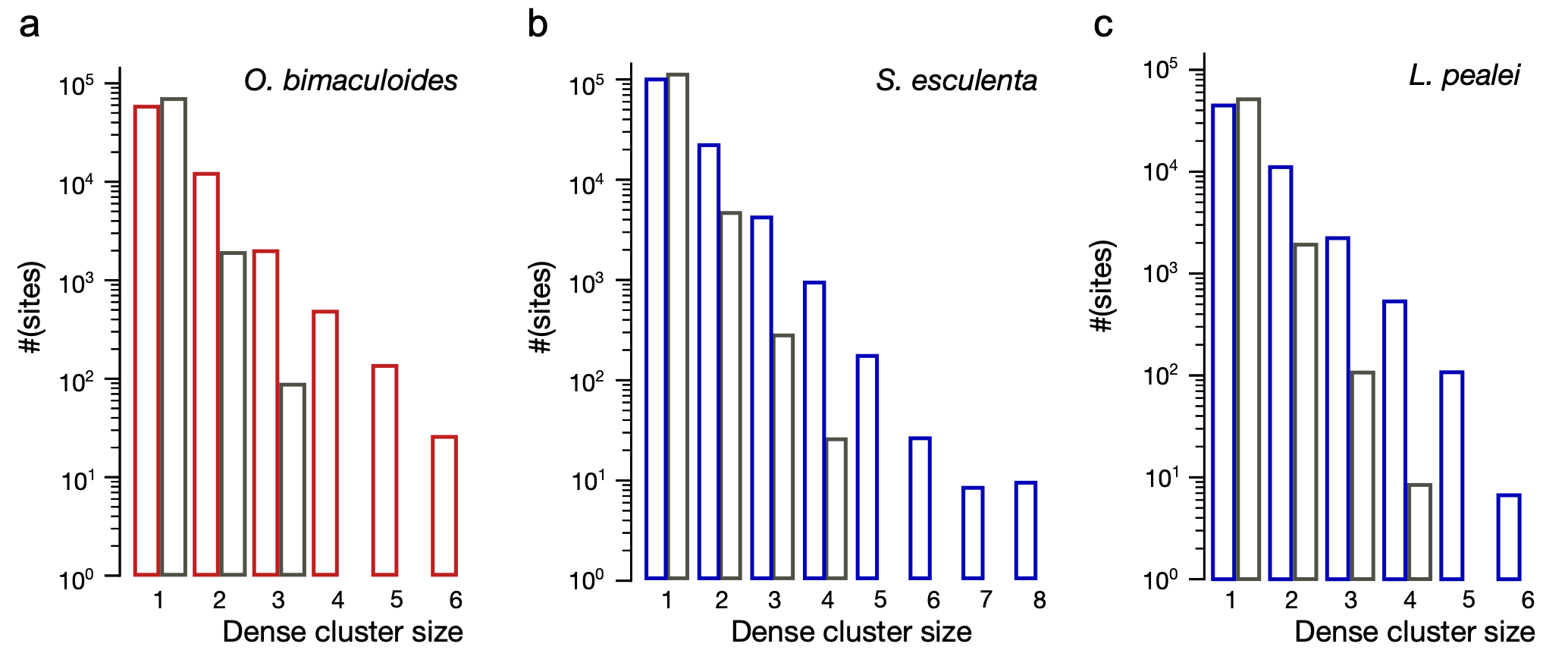


**Supplementary Figure S5 | Histograms of dense cluster sizes (nt) for the real coleoid editing site datasets (red and blue) and the corresponding randomly obtained ones (grey).** **(a)** *O. bimaculoides* **(b)** *S. esculenta* **(c)** *L. pealei*. Notation as in Fig. 3A.


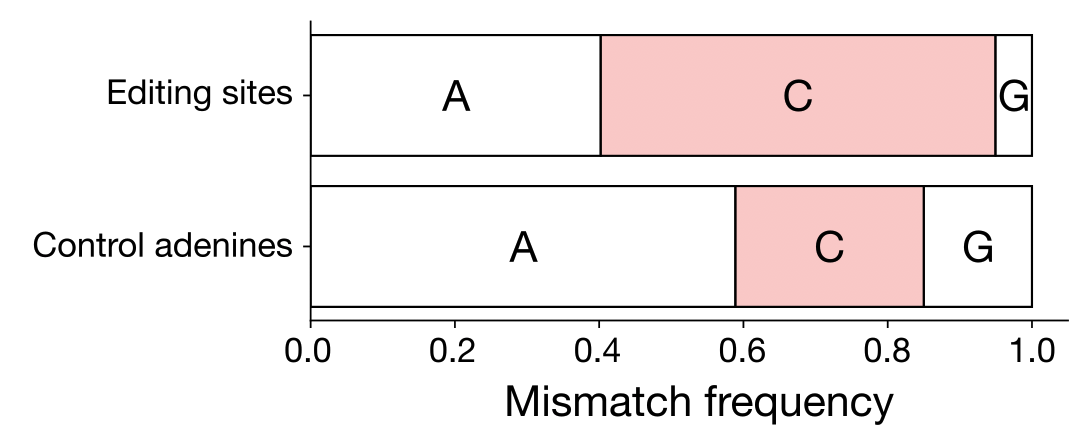


**Supplementary Figure S6 | Distributions of mismatches of adenines in double RNA helices.** See Supplementary Methods for details.


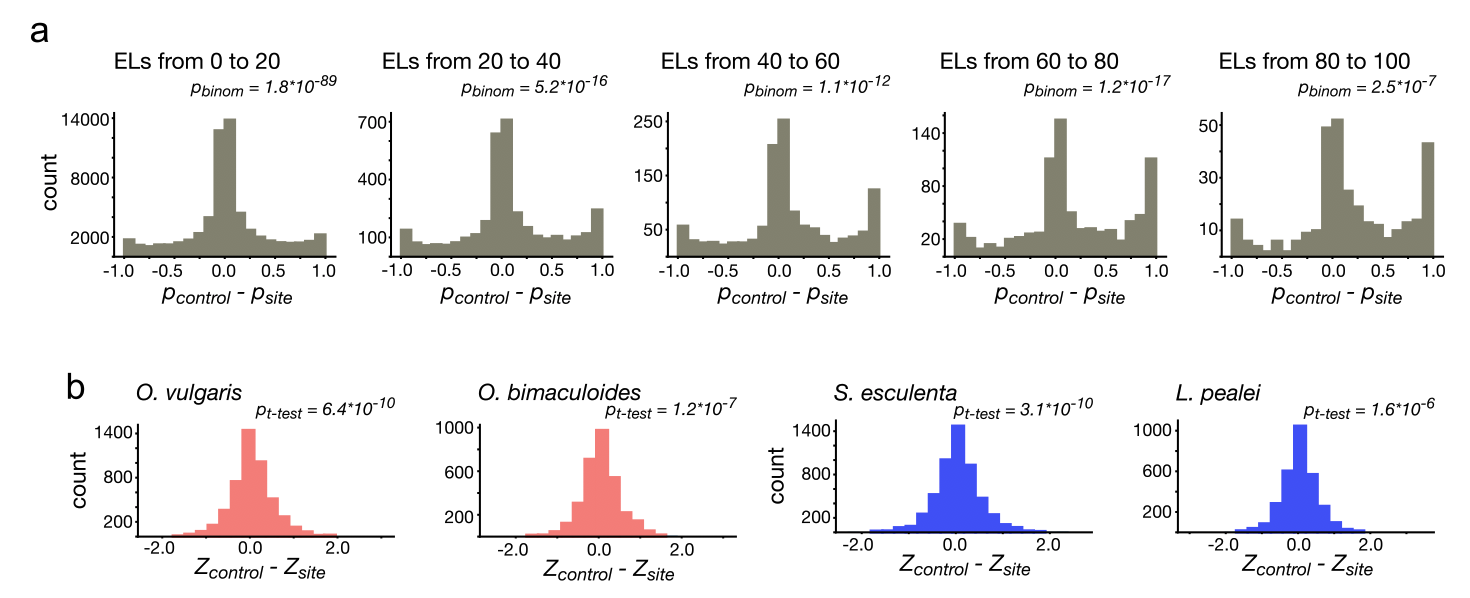


**Supplementary Figure S7| Structural properties of coleoid A-to-I editing sites. (a)** Differences in base pairing probabilities between A-to-I editing sites and nearest unedited adenines used as controls for different editing level (EL) intervals in *O. vulgaris*. The significance (*p*-values, the binomial test) of the site–control differences is shown. **(b)** Differences in RNA secondary structure free energy between editing sites and control adenines in four coleoid species. The significance (*p*-values, the *t*-test) of the control sites structural potentials being larger than those of editing sites is shown.

**
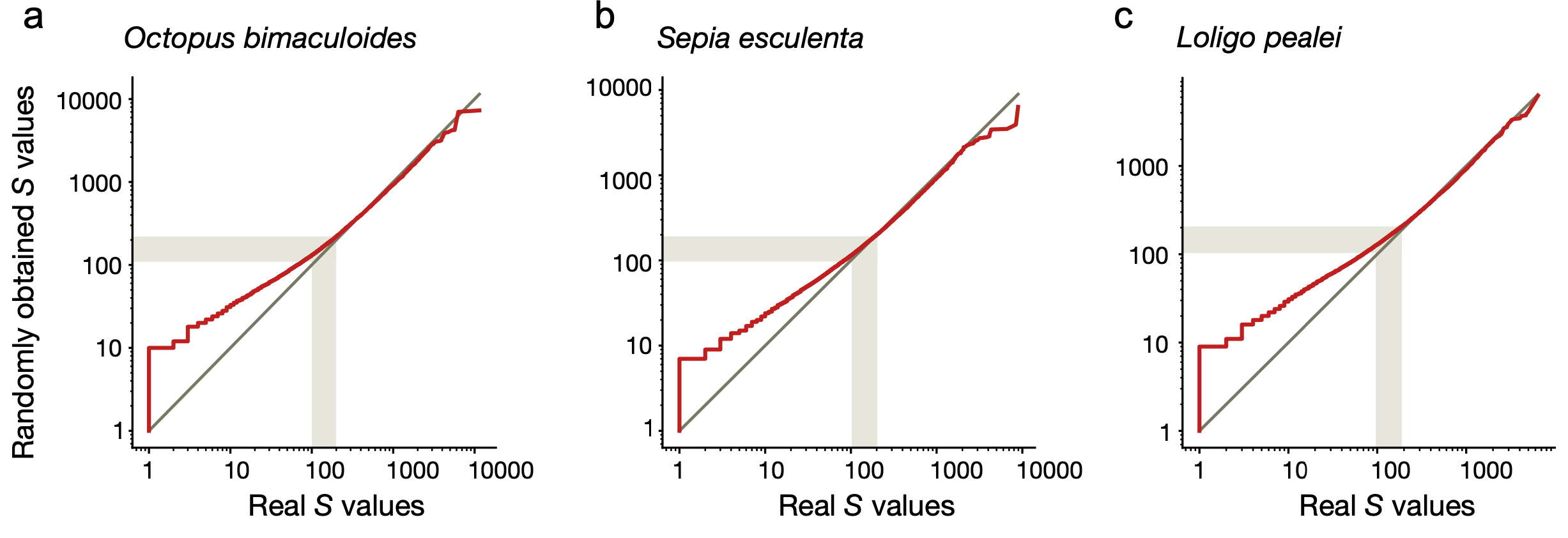
**

**Supplementary Figure S8 | Clustering of A-to-I editing sites in coleoid transcriptomes.** Red lines show the dependencies between the sorted real and the randomly obtained *S* value sets. Grey lines represent the expected dependence of the form *y=x*. Grey stripes represent the predicted borders of the regions affecting editing sites. **(a)** *O. bimaculoides* **(b)** *S. esculenta* **(c)** *L. pealei*. Notation as in Fig. 5A.


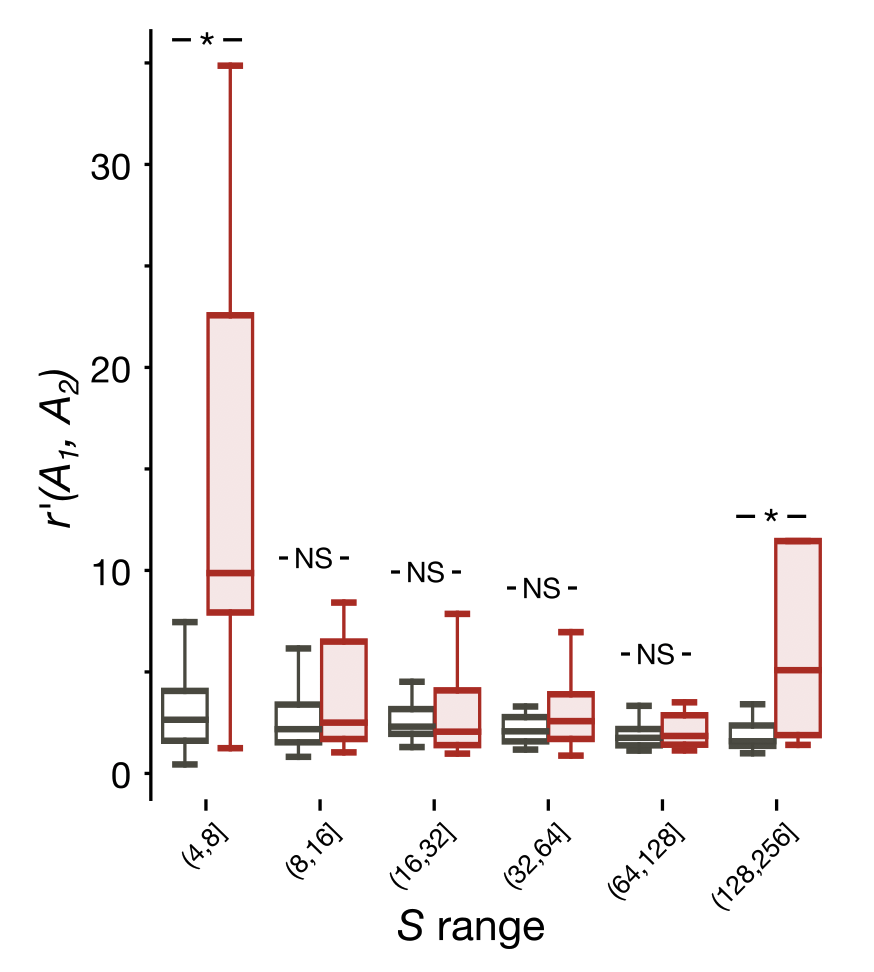


**Supplementary Figure S9 |** Distributions of the $r^{'}\left( A_{i},A_{j} \right)$ values calculated for the structurally close editing sites (red boxes) and for the control site pairs with no predicted secondary RNA structure between the sites in a pair (grey boxes), where both sites $A_{i}$ and $A_{j}$ are located in the same exon of *O. bimaculoides***.** Notation as in Fig. 5B.


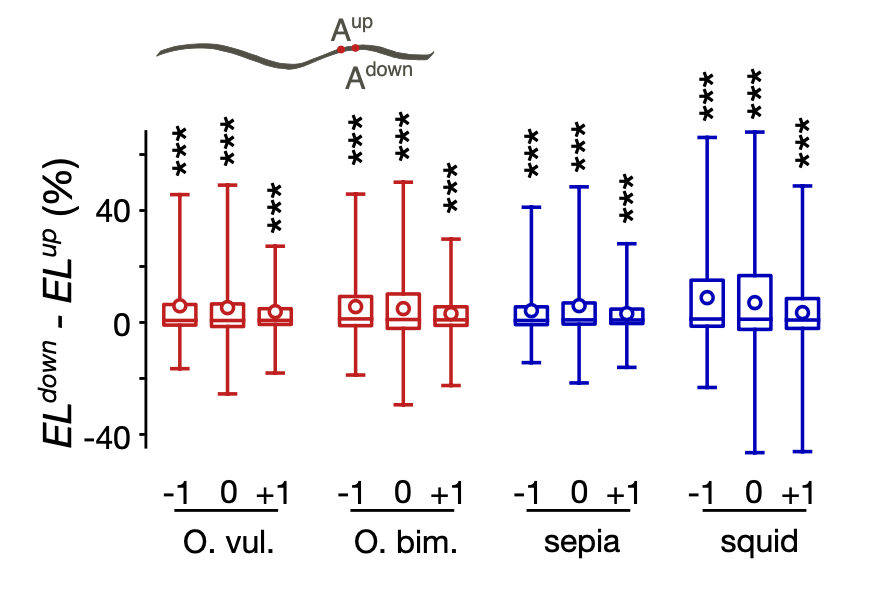


**Supplementary Figure S10 | Distributions of differences in ELs between down- and upstream editing site in two-adenine dense clusters obtained for three reading frames.** Three asterisks mark statistical significance of the differences in means (*p* < 0.001, Chi-squared contingency test). The values -1, 0 and +1 below indicate the coordinate of the dense cluster reading frame relative to the predicted protein-coding frames in the coleoid transcriptomes.

**Supplementary table S1 | SRA identifiers of RNAseq libraries employed in the present study**

| **SRA ID** | **Organism** | **Bioproject** | **#G bases** |
| --- | --- | --- | --- |
| SRR2045866 | *O. bimaculoides* | PRJNA285380 | 5.6 |
| SRR2045870 | *O. bimaculoides* | PRJNA285380 | 7.4 |
| SRR2047107 | *O. bimaculoides* | PRJNA285380 | 7.2 |
| SRR2047109 | *O. bimaculoides* | PRJNA285380 | 7.9 |
| SRR2047111 | *O. bimaculoides* | PRJNA285380 | 7.1 |
| SRR2047114 | *O. bimaculoides* | PRJNA285380 | 6.8 |
| SRR2047116 | *O. bimaculoides* | PRJNA285380 | 6.4 |
| SRR2047118 | *O. bimaculoides* | PRJNA285380 | 6.1 |
| SRR2047120 | *O. bimaculoides* | PRJNA285380 | 6.3 |
| SRR2047122 | *O. bimaculoides* | PRJNA285380 | 7.2 |
| SRR2048495 | *O. bimaculoides* | PRJNA285380 | 3.2 |
| SRR2048496 | *O. bimaculoides* | PRJNA285380 | 3.2 |
| SRR2048497 | *O. bimaculoides* | PRJNA285380 | 3.2 |
| SRR2048498 | *O. bimaculoides* | PRJNA285380 | 3.2 |
| SRR2048521 | *O. bimaculoides* | PRJNA285380 | 3.2 |
| SRR2048522 | *O. bimaculoides* | PRJNA285380 | 3.2 |
| SRR2048523 | *O. bimaculoides* | PRJNA285380 | 3.2 |
| SRR2048524 | *O. bimaculoides* | PRJNA285380 | 3.2 |
| SRR2048525 | *O. bimaculoides* | PRJNA285380 | 3.2 |
| SRR2857272 | *O. vulgaris* | PRJNA299756 | 38.2 |
| SRR2857274 | *O. vulgaris* | PRJNA299756 | 38.4 |
| SRR2855904 | *S. esculenta* | PRJNA299756 | 39.6 |
| SRR2856422 | *S. esculenta* | PRJNA299756 | 48.3 |
| SRR1522987 | *L. pealei* | PRJNA255916 | 17.5 |
| SRR1522988 | *L. pealei* | PRJNA255916 | 17.5 |
| SRR1725163 | *L. pealei* | PRJNA255916 | 6.9 |
| SRR1725164 | *L. pealei* | PRJNA255916 | 6.9 |
| SRR1725167 | *L. pealei* | PRJNA255916 | 5.6 |
| SRR1725169 | *L. pealei* | PRJNA255916 | 7.7 |
| SRR1725171 | *L. pealei* | PRJNA255916 | 5.6 |
| SRR1725172 | *L. pealei* | PRJNA255916 | 4.1 |
| SRR1725213 | *L. pealei* | PRJNA255916 | 10.8 |
| SRR1725235 | *L. pealei* | PRJNA255916 | 12.4 |
| SRR1725236 | *L. pealei* | PRJNA255916 | 8.3 |

**Supplementary Table S2 | Variance in the transcriptome and proteome explained by editing and by correlations in editing events**

|  | **O. vul.** | **O. bim.** | **sepia** | **squid** |
| --- | --- | --- | --- | --- |
| **nucleotide SD due to editing** | 97.3027 | 82.2 | 110.8405 | 94.5532 |
| **% nucleotide variance explained by clustering** | 40.34 | 27.69 | 46.28 | 31.59 |
| **amino acid SD due to editing** | 80.6424 | 68.39 | 92.008 | 78.88 |
| **% amino acid variance explained by clustering** | 40.69 | 28.46 | 46.5 | 32.34 |
| **% nucleotide variance explained by DCs** | 4.3 | 3.6 | 4.038 | 4.057 |
| **% amino acid variance explained by DCs** | 4.48 | 3.7 | 4.168 | 4.175 |

**Supplementary Table S3 | Effect sizes and confidence intervals for the data presented on Fig. 6C.** Red color of a letter indicates the nucleotide in a dinucleotide, for which base-pairing probabilities are considered. *p_1_ – p_2_* represents the mean difference in base pairing probabilities.

| **Comparison** | ***p_1_ - p_2_*** | **Wilcoxon p (Bonferroni corrected)** |
| --- | --- | --- |
| EE vs AA | -0.0268 | 1.0×10^–6^ |
| EE vs AA | 0.11917 | 2.8×10^–72^ |
| EE vs AA | -0.1399 | 3.8×10^–109^ |
| EE vs AA | 0.00603 | 0.8475 |
| EE vs AE | -0.0776 | 4.0×10^–16^ |
| EE vs AE | 0.03575 | 0.0011 |
| EE vs AE | -0.1826 | 1.1×10^–70^ |
| EE vs AE | -0.0693 | 2.7×10^–13^ |
| EE vs EA | 0.07293 | 8.4×10^–6^ |
| EE vs EA | 0.17896 | 4.5×10^–24^ |
| EE vs EA | -0.0519 | 0.0022 |
| EE vs EA | 0.05410 | 0.0017 |

**Supplementary Table S4 | 95% Confidence intervals of differences in base-pairing probabilities between paired editing sites (EE) and three types of control AA-dinucleotides obtained by random sampling.** The color code is as in Table S3.

| **position \ site pair** | **AA - EE** | **AE - EE** | **EA - EE** |
| --- | --- | --- | --- |
| **1** | 0.0139 to 0.0396 | 0.0573 to 0.0968 | -0.1057 to -0.0399 |
| **2** | -0.0187 to 0.0072 | 0.0494 to 0.0896 | -0.0881 to -0.02 |
